# Developmentally Regulated Oscillations in the Expression of UV Repair Genes in a Soilborne Plant Pathogen Dictate UV Repair Efficiency and Survival

**DOI:** 10.1128/mBio.02623-19

**Published:** 2019-12-03

**Authors:** Shira Milo-Cochavi, Sheera Adar, Shay Covo

**Affiliations:** a Department of Plant Pathology and Microbiology, Robert H. Smith Faculty of Agriculture Food and Environment, Hebrew University of Jerusalem, Rehovot, Israel; b Department of Microbiology and Molecular Genetics, The Institute for Medical Research Israel-Canada, Faculty of Medicine, Hebrew University of Jerusalem, Jerusalem, Israel; Tel Aviv University

**Keywords:** DNA repair, *Fusarium oxysporum*, UV

## Abstract

The ability to withstand UV damage shapes the ecology of microbes. While mechanisms of UV tolerance were extensively investigated in microorganisms regularly exposed to the sun, far less is known about UV repair of soilborne microorganisms. Fusarium oxysporum is a soilborne fungal plant pathogen that is resistant to UV light. We hypothesized that its UV repair capacity is induced to deal with irregular sun exposure. Unlike the SOS paradigm, our analysis revealed only sporadic increases and even decreases in UV repair gene expression following UVC irradiation or exposure to visible light. Strikingly, a major factor determining the expression of UV repair genes was the developmental status of the fungus. At the early stages of germination, the expression of photolyase increased while the expression of UV endonuclease decreased, and then the trend was reversed. These gene expression oscillations were dependent on cell cycle progression. Consequently, the contribution of photoreactivation to UV repair and survival was stronger at the beginning of germination than later when a filament was established. F. oxysporum germinates following cues from the host. Early on in germination, it is most vulnerable to UV; when the filament is established, the pathogen is protected from the sun because it is already within the host tissue.

## Abstract (precis)

Fusarium oxysporum infects plants through the roots and therefore is not exposed to the sun regularly. However, the ability to survive sun exposure expands the distribution of the population. UV from the sun is toxic and mutagenic, and to survive sun exposure, fungi encode several DNA repair mechanisms. We found that Fusarium oxysporum has a gene expression program that activates photolyase at the first hours of germination when the pathogen is not established in the plant tissue. Later on, the expression of photolyase decreases, and the expression of a light-independent UV repair mechanism increases. We suggest a novel point of view to a very fundamental question of how soilborne microorganisms defend themselves against sudden UV exposure.

## INTRODUCTION

UV light is probably the most important environmental genotoxic agent. Microorganisms that are exposed to strong UV radiation, such as marine *Flavobacteriia* or Acinetobacter strains that live at high altitude, show diverse and efficient UV repair or damage avoidance mechanisms (1, 2). Far less is known about the DNA damage response of microorganisms that are not regularly exposed to UV. Escherichia coli is the model organism to study the repair of UV damage in organisms that are not exposed constantly to light. It has a sophisticated mechanism to induce the expression of UV repair genes known as the SOS system that may contribute to its ability to survive in diverse ecological niches (3–5). Fusarium oxysporum, the focus of this work, is an economically important fungal plant pathogen; one of the subspecies of this fungus endangers banana growth worldwide (6–8). F. oxysporum is a soilborne pathogen that penetrates plants through their roots. Therefore, no sun exposure is expected during most or even all of its life cycle. Nevertheless, F. oxysporum can survive high doses of UV (9). F. oxysporum has three types of spores (10–12). Microconidia are probably the most common type of F. oxysporum spore. Microconidia are root propagules that are found in the soil, although their occasional exposure to the sun is possible. The two other types are chlamydospores and macroconidia. Chlamydospores are heavily melanized resting units that can survive in the ground for years (10). These spores are expected to be UV resistant. Macroconidia are multinucleate spores that are usually formed on plant stems or leaves (10). Here, we focus our analysis on microconidia; the relevance of our results to other types of spores is discussed. Conidia germinate in response to plant signals, such as amino acids and plant peroxidases, but also in response to humidity and other nutrients (13, 14). Taking the disease cycle of F. oxysporum into account, the UV response of the fungus can be divided in two; the UV damage induction probably occurs during conidial germination, but development of the germlings into hyphae probably occurs already within the plant tissue and therefore away from UV and visible light (15).

The most common UV lesions are cyclobutane pyrimidine dimers (CPDs) and 6,4 UV photoproducts, both of which significantly distort the DNA helix. This severe disruption of the DNA structure interrupts DNA replication and transcription, thereby threatening the survival of microbes. UV is also mutagenic; it may cause deleterious mutations in the surviving population. Microorganisms have developed several mechanisms to deal with UV exposure (16–18). Ascomycete filamentous fungi are known for their remarkable ability to sustain UV exposure due to the function of three DNA repair mechanisms, nucleotide excision repair (NER), UV damage endonuclease (UVDE), and photolyase (19–21). NER does not recognize UV specifically; rather, it recognizes distortion of the DNA helix by scanning the genome (global NER) using the proteins Ddb1 and Xpc (22–24). Alternatively, the lesions are recognized by stalled RNA polymerase II with the assistance of proteins Csa and Csb, a process known as transcription-coupled NER (22, 23, 25, 26). After damage detection, Xpa, Xpb, and Xpd facilitate incisions on both sides of the lesion by two nucleases, Xpg and Xpf. The oligonucleotide containing the lesion is removed, and the gap is filled by DNA polymerases (22, 25). UVDE directly recognizes the two major UV lesions and makes a nick in the 5ʹ direction from them that is then further processed (27, 28). Photolyases are found throughout evolution, except in placental mammals. Like UVDE, photolyases also bind UV lesions directly, but the repair mechanism is very different. Photolyases are very specific to UV lesions, with the ascomycete enzymes only binding CPDs (29, 30). The photolyase protein Phr1 repairs UV dimers using photon energy from the blue end of sunlight (in the laboratory, UVA light is used to activate the enzyme). Thus, Phr1-dependent repair is considered to be photoreactivation-dependent repair, whereas the other UV repair mechanisms are known as dark repair. Reports from E. coli and Saccharomyces cerevisiae also suggest a role for Phr1 in NER by guiding the machinery to the site of the UV lesions (31).

A comprehensive SOS-like response to UV has never been demonstrated in fungi. Nevertheless, some DNA repair/DNA damage tolerance genes have been shown to be induced by UV in S. cerevisiae, Neurospora crassa, and Schizosaccharomyces pombe (32, 33). Another environmental inducer of DNA repair proteins is light. Fungi respond strongly to light, which affects diverse aspects of their biology (34–36). Visible/blue light has been shown to induce UV repair genes, which is correlated with increased repair capacity (29, 37–40). In the fungus Cryptococcus neoformans, *uvde* has been shown to be regulated by the white collar complex, an important light response determinant. This regulation increases the ability of fungi to survive UVC radiation (37).

All UV repair pathways described here (NER, photolyase, and UVDE) are considered prereplication repair pathways in that they act before the replication fork encounters the UV lesion and thus prevent replication fork arrest by the lesion. Bypassing lesions that escape prereplication repair is often a mutagenic process (41–43). In filamentous fungi, the first nuclear replication during conidial germination is very important, because if a mutation occurs at this stage, it will affect the entire hyphae. Therefore, it would be most beneficial if the first cell division cycles had the highest possible repair capacity.

Due to the soilborne lifestyle of F. oxysporum, we hypothesized that its response to UV irradiation would be inducible. However, using quantitative PCR (qPCR) gene expression analysis, we revealed no clear induction pattern of NER, *phr1*, or *uvde*. Instead, we revealed considerable changes in the expression of *phr1* and *uvde* during cell cycle progression and germling development. At first, when germlings contain mainly nuclei in the S phase, the expression of *phr1* is induced and that of *uvde* is reduced. Later, when the germling matures into a hypha and the number of S-phase nuclei decreases, the trend is reversed. We were able to attenuate the expression changes using drugs that block cell cycle progression. Finally, we were able to show that photoreactivation-assisted UV repair is indeed induced when *phr1* transcripts are induced. We discuss the meaning of our results in the ecological and evolutionary context of *Fusarium* wilt disease.

## RESULTS

### UV repair genes exhibit a complex transcriptional response to UV, visible light, and sunlight.

To study the response of F. oxysporum to UV either from a germicidal lamp or the sun, we performed RNA sequencing (RNA-seq) analysis. We used the Lexogen 3′ QuantSeq kit in which only the 3′ end of the gene is sequenced, allowing cost-effective analyses of major trends in gene expression changes across multiple conditions. For accurate determination of the expression of specific genes, we used quantitative PCR, as detailed below. To study the effect of UVC on gene expression, fungi were irradiated at two developmental stages, at 8 and 14 h postinoculation. During 8 h postinoculation, only one or two rounds of replication occur. At 14 h postinoculation, the entire population broke dormancy, and several rounds of the nuclear division had occurred. This is why the analysis of 14 h postinoculation was more detailed. Importantly, from ecological standpoint, 8 h postinoculation resembles the status of the pathogen during early stages in germination and infection, while 14 h postinoculation resembles elongation of the filament and establishment within the root tissue. As described below, the developmental stage of the fungus played a significant role in the response to UV. The different conditions of the RNA-seq analyses are presented in Table 1. The up- and downregulated genes in the different treatments are described in Table S2 in the supplemental material. We analyzed Gene Ontology (GO) terms for over- and underexpressed genes following UV irradiation in both developmental stages. DNA repair was not identified as an enriched module among the overexpressed genes in any of our treatments (Text S1). The GO terms that were enriched following UV exposure are summarized in Text S1. The most significant GO term for upregulated genes at 14 h postinoculation was translation (*P* = 10^−65^), and the most significant GO term for downregulated genes at 14 h postinoculation was oxidoreductases (*P* = 10^−4^). The most significant GO term for upregulated genes at 8 h postinoculation was transferase activity (*P* = 10^−3^), and the most significant GO term for downregulated genes at 8 h postinoculation was RNA processing (*P* = 10^−27^). We observed opposite trends in response to UV; GO terms that were upregulated in response to UV when 14 h germlings were irradiated were downregulated when 8 h germlings were treated (Text S1). For example, noncoding RNA processes were upregulated following irradiation at 14 h (*P* = 10^−21^) and downregulated following irradiation at 8 h (*P* = 10^−18^). As discussed in detail below, we think that this phenomenon is due to the basal uninduced level of expression at the different developmental stages.

**TABLE 1:** RNA-seq analyses^*a*^

Time postinoculation before irradiation (h)	Treatment	Time of incubation following irradiation (m)
14	50 J/m^2^	0
14	50 J/m^2^	30
14	50 J/m^2^	60
14	200 J/m^2^	0
14	200 J/m^2^	30
14	200 J/m^2^	60
8	50 J/m^2^	30
8	200 J/m^2^	30
14	2 h sunlight	

a The different conditions used for RNA-seq experiments are described. The results are presented in Table S2 and summarized in Text S1 and S2 for all except the 2 h in sunlight (shown in Text S3).

10.1128/mBio.02623-19.6 TABLE S2 RNA sequencing results. Fold increases in the expression of genes following UV and sun exposures are shown. The sequencing experiments were done twice, with three different biological replicates in each of them. Download Table S2, XLSX file, 11.2 MB. Copyright © 2019 Milo-Cochavi et al. 2019 Milo-Cochavi et al. This content is distributed under the terms of the Creative Commons Attribution 4.0 International license.

We next examined the expression of genes that are directly involved in UV DNA damage repair. When 14 h-postinoculation germlings were irradiated with 50 J/m^2^ UV, an equal number of DNA repair genes were modestly upregulated and downregulated; at 200 J/m^2^, the expression of UV repair genes decreased (Text S2). The picture was different when 8 h-postinoculation germlings were irradiated. Clear induction of some NER-related genes was observed especially at 50 J/m^2^, for example, *rad1*, *rad14*, *rad2*, *rad4*, and *xpc* (Text S2). Next, we measured the transcriptomic response to 2 h of sunlight exposure at midday (12:00 to 14:00) on a sunny day during the summer in Israel (12 July 2017). The up- and downregulated genes are described in Table S2. GO term analysis did not show significant induction of DNA repair, but it did show induction of genes related to oxidative stress (*P* = 0.02) (Text S3). Heat map analysis of UV repair genes did not show clear induction by sunlight, and there was a moderate induction of genes related to the circadian clock, such as *wc-1* (Text S3).

**FIG 1:**
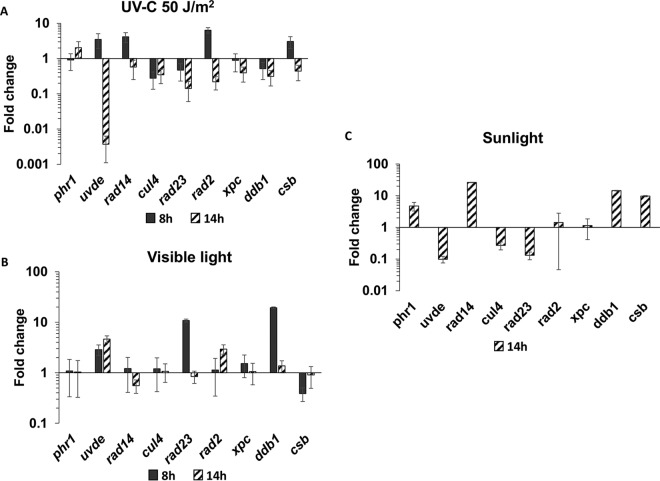
Mixed trends in the effects of UV, visible light, and solar radiation on the expression of UV repair genes. Real-time quantitative PCR analysis of the expression of several UV repair genes was performed at 8 and 14 h postinoculation. (A) 50 J/m^2^ UV followed by 30 min recovery; *ef1-a* served as a reference gene. (B) Ten minutes of exposure to fluorescent light; *gapdh* served as a reference gene. (C) Two hours in the sun (see the text and Materials and Methods; *gapdh* served as a reference gene). In all panels, dark-gray bars indicate 8 h postinoculation, while striped bars indicate 14 h postinoculation.

We measured the expression of individual UV repair genes using quantitative PCR of conidia that were inoculated in potato dextrose broth (PDB) medium either for 8 or 14 h. Several conditions were measured, as follows: (i) UVC, (ii) 10-min exposure to fluorescent light, and (iii) 14 h exposure to solar radiation (Table 1). These analyses clearly showed a change in the expression of several UV repair genes. UV irradiation at 50 J/m^2^ of 14 h postinoculation germlings reduced the expression of most tested genes, including a 100-fold drop in the expression of *uvde* (Fig. 1A). Induction by UVC of 5- to 10-fold was observed in *uvde*, *rad14*, *rad2*, and *csb* when germlings were irradiated at 8 h postinoculation (Fig. 1A). Exposure to visible light resulted in a 10- to 20-fold induction of *uvde*, *rad23*, and *ddb1* (Fig. 1B). A ca. 10-fold induction of *phr1*, *ddb1*, and *csb* was observed when germlings were exposed to sunlight at 14 h postinoculation (Fig. 1C), in agreement with previous results (9, 40). The effect of UVC on both UV-specific genes *phr1* and *uvde* was highly dependent on the timing of irradiation; we suspected that this was due to the baseline levels of the transcripts at the different time points postinoculation.

### Oscillations in the expression of UV repair genes during fungal development.

**FIG 2:**
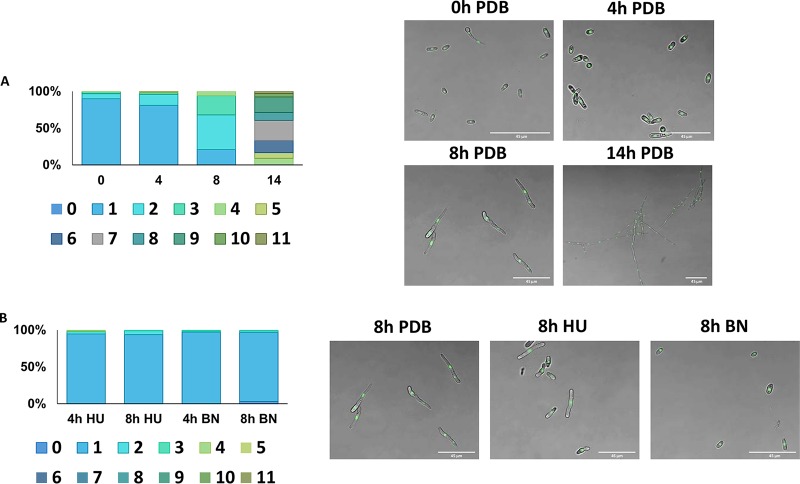
The number of nuclei in germlings 8 and 14 h postinoculation suggests differences in cell cycle profiles. (A) The number of nuclei at 0, 4, 8, and 14 h postinoculation in 160, 100, 100, and 66 germinating conidia, respectively, was determined by analyzing microscope images of a histone H1-GFP strain. The color-coded squares represent the number of nuclei/germlings. (B) Same in panel A, but germinating conidia were treated with 200 mM HU for 4 or 8 h (95 and 75 germlings, respectively) or 50 μg/ml benomyl (BN) for 4 or 8 h (100 germlings).

The different effects of UV and visible light on gene expression at different time points postinoculation lead us to hypothesize that the cell cycle status of the germling is an important determinant in the basal expression level of UV repair genes. It was previously shown that during the germination and germ tube elongation process of F. oxysporum, only one nucleus is mitotically active, while all the rest are dormant (44). Therefore, while at 8 h postinoculation the ratio between mitotic active and dormant nuclei is expected to be high, at 14 h postinoculation, most of the nuclei are expected to be dormant. Using a histone 1-green fluorescent protein (H1-GFP) fusion encoded by the native locus, we were able to count the number of nuclei at 0, 4, 8, and 14 h postinoculation (Fig. 2A). The populations were significantly different regarding the number of nuclei/germling (*P* = 2 × 10^−16^). The number of nuclei/germling at 14 h postinoculation was much higher than at 8 h postinoculation. For example, 47% of the population of 8-h-postinoculated germlings contained only 2 nuclei. We could not detect germlings with 2 nuclei at 14 h postinoculation (Fig. 2A). In contrast, while we could not identify germlings with 6 nuclei or more at 8 h postinoculation, 76% of the 14-h-postinoculation population contained 6 or more nuclei (Fig. 2A). We were also able to show that we could arrest nucleus duplication by using the DNA replication inhibitor hydroxyurea or the mitosis inhibitor benomyl. In both cases, at 8 h postinoculation, the proportion of 2 nuclei germlings from the total population was significantly reduced compared to that with uninterrupted growth (Fig. 2B).

**FIG 3:**
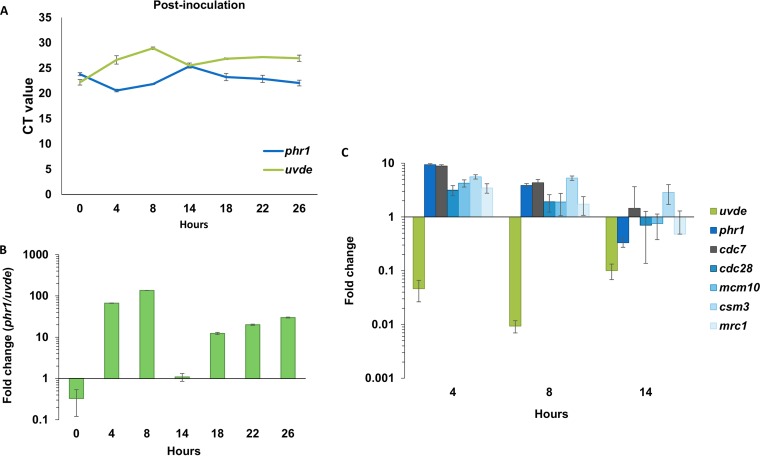
Opposite oscillations in *phr1* and *uvde* expression correlate the expression of cell cycle genes controlled by cell cycle progression. Quantitative PCR analysis of *uvde* and *phr1* was performed at several time points postinoculation using microconidia in PDB medium. (A) Threshold cycle (*C_T_*) values of *phr1* and *uvde* at different time points postinoculation in PDB medium. (B) Ratio of *phr1* to *uvde* expression (as expressed by the inverse ratio of their quantitative PCR *C_T_* values) was determined at several time points between 0 and 26 h postinoculation. (C) Changes in the expression of *phr1*, *uvde*, and cell cycle-regulated genes (*cdc7*, *cdc28*, *mcm10*, *mrc1*, and *csm3*) during the development of germinating conidia. Fold change values were calculated in comparison with the expression in resting conidia.

We hypothesized that the expression of the UV repair genes was altered during the germling development due to the ratio of mitotically active and dormant nuclei. Using quantitative reverse transcription-PCR (RT-qPCR), we measured the expression of *phr1* and *uvde* in untreated conidia from the time of inoculation to 26 h postinoculation. We observed a periodic pattern of expression. In the first 8 h, the expression of *phr1* and *uvde* showed a 5- to 8-fold increase and 16- to 64-fold decrease, respectively; from 8 to 14 h, the trend reversed. From 14 h to 26 h postinoculation, there was another reduction in *uvde* expression and induction in the expression of *phr1* (Fig. 3A). Unlike *phr1* and *uvde*, there was no clear oscillating pattern in the expression of other DNA repair genes such as the repair genes induced by methyl methanesulfonate (MMS) damage (*mgt1* and *mag1*) or NER (*rad14*, *rad2*, *rad23*, and *cul4*) (Text S4). Some changes in expression were observed for *xpc* and *ddb1*, but they were not as pronounced as the changes in *uvde* and *phr1* expression (Text S4).

### Cell cycle regulation of oscillations in *phr1* and *uvde* expression.

The phenomenon of opposite oscillations in *phr1* and *uvde* expression is interesting because these two enzymes compete for specific binding to the same lesion, but their mechanisms of repair are very different. Whereas Phr1 is only activated during exposure to visible light (including UVA), Uvde can repair UV-induced lesions without light, i.e., after the fungus penetrates the plant tissue or is relocated to the root habitat. Therefore, we calculated the RNA expression ratio of *phr1* to *uvde* (*phr1*/*uvde*). In resting conidia, the *phr1*/*uvde* ratio was close to 1, while at 8 h postinoculation, it was 100 and returned to a value of 1 at 14 h postinoculation (Fig. 3B).

**FIG 4:**
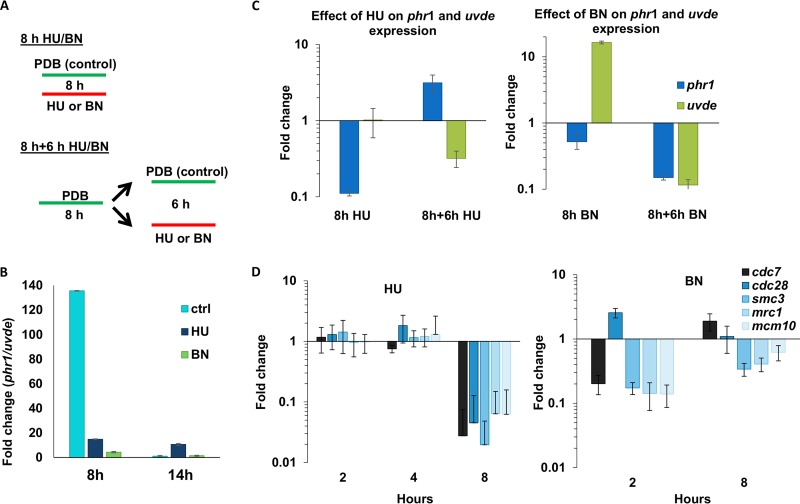
Opposite oscillations in *phr1* and *uvde* expression are controlled by cell cycle progression. (A) The ratio of *phr1* to *uvde* expression was determined in two experimental scenarios, as follows: (i) 8 h postinoculation in PDB containing 200 mM HU or 10 μg/ml benomyl (BN); and (ii) 14 h postinoculation, where for the first 8 h, conidia were grown in PDB, and then 200 mM HU or 10 μg/ml BN was added for another 6 h. (B) The ratio of the expression of *phr1* and *uvde* following HU and BN exposures as described in panel A. (C) The effect of HU and BN on the expression of *phr1* and *uvde* in the experimental setting described in panel A. The fold increase between treated to untreated was calculated using the ΔΔ*C_T_* method. For both HU and BN exposures, the normalizing gene was actinL binding protein. (D) The effect of HU and BN on the expression of cell cycle-regulated genes (*cdc7*, *cdc28*, *mcm10*, *mrc1*, and *csm3*) during the development of germinating conidia (2 to 8 h postinoculation). Fold increase was calculated using the ΔΔ*C_T_* method of treated versus untreated.

Developmental biology in fungi is highly dependent on cell cycle progression. At the first 14 h postinoculation, the expression of *phr1* showed the same trend as several cell cycle and DNA replication genes such as *cdc7*, *mcm10*, and *cdc28* (Fig. 3C). In comparison with resting conidia, the expression of these cell cycle genes is increased in the first 8 h; at 14 h postinoculation, the increase is attenuated (Fig. 3C). In contrast, the expression of *uvde* is decreased in the first 8 h (Fig. 3C). To determine whether the changes in gene expression of *uvde* and *phr1* are controlled by the cell cycle and not merely correlated with it, we measured the expression of these genes under conditions of cell cycle arrest. Two experimental settings were used (Fig. 4A). In the first, conidia were inoculated for 8 h with hydroxyurea (HU) or the M-phase inhibitor benomyl (Fig. 4A). As a control, conidia were inoculated for 8 h without cell cycle inhibition. The ratio between *phr1* and *uvde* was 10 times lower after exposure to 200 mM HU, compared to that with uninterrupted growth (Fig. 4B). HU exposure caused a ca. 10-fold reduction in *phr1* expression and changed the amount of *uvde* only modestly (Fig. 4C). When cells were exposed to 10 μg/ml benomyl, the ratio of *phr1* to *uvde* was also significantly lower than in the control (Fig. 4B) due to a 16-fold increase in the amount of *uvde* when the benomyl was added (Fig. 4C). The second experimental setting was inoculation of conidia for 8 h in PDB medium and then the addition of either HU or benomyl for another 6 h. As a control, no inhibitor was added. When 200 mM HU was added to the culture 8 h after inoculation, the *phr1*/*uvde* ratio was much higher than under uninterrupted growth (Fig. 4B) due to an almost 10-fold increase in the amount of *phr1* transcript and a 3-fold reduction in the amount of *uvde* (Fig. 4C). When 10 μg/ml benomyl was added to the medium 8 h after inoculation, the *phr1/uvde* ratio was similar to that in the control, but the expression of both genes was reduced by ca. 10-fold (Fig. 4C). For the most part, DNA replication and cell cycle genes responded to HU and benomyl at 8 h postinoculation, similarly to *phr1* (Fig. 4D). In conclusion, halting hyphal development through cell cycle arrest disrupted the alterations in the expression of *phr1* and *uvde*.

### UV repair efficiency is tightly correlated with oscillations in the expression of UV repair genes.

**FIG 5:**
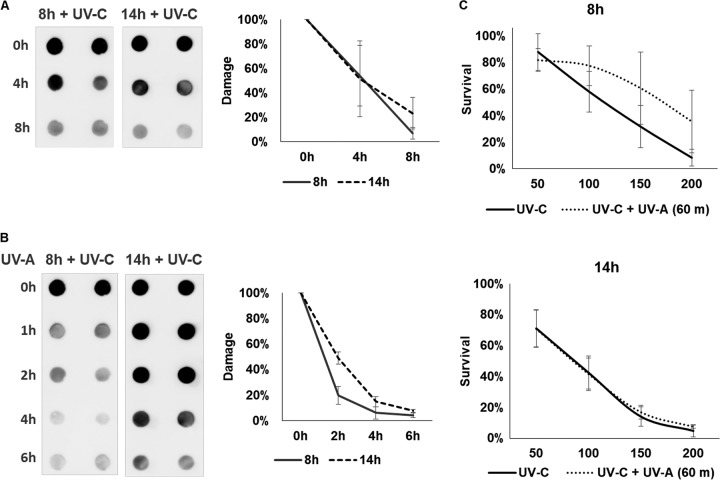
Photoreactivation-assisted UV repair and survival are enhanced when cells are irradiated 8 h postinoculation than at 14 h postinoculation. (A) A UV repair assay. Conidia were inoculated in PDB for either 8 or 14 h, and then cells were irradiated with 50 J/m^2^ UVC. After irradiation, cells were incubated at room temperature for the indicated time periods without UVA exposure (see Materials and Methods). Then, the number of UV lesions was determined by immunodot blot assay using antibodies against CPD (see Materials and Methods). The strength of the immunodot blot signal is shown as the percentage of lesions left out of the initial damage after the incubation periods. (B) Same as in panel A, but this time, UVA exposure was done for the indicated time periods. (C) UV survival assay. About 200 to 500 CFU either 8 or 14 h postinoculation were spread on PDA plates and irradiated (0, 50, 150, and 200 J/m^2^ UVC). Plates were then incubated at room temperature for 1 h with or without UVA exposure (see Materials and Methods). Survival was calculated as the number of CFU after irradiation out of the number of CFU without irradiation.

To examine whether the differential expression of *phr1* and *uvde* during fungal development affects DNA repair *per se*, we measured the repair of cyclopyrimidine dimers (CPDs) facilitated by UVA light. Phr1 binds CPDs in a light-independent manner; however, photolyase activity is dependent on UVA light (photoreactivation). We inoculated conidia for 8 and 14 h, and then the fungi were irradiated with 50 J/m^2^ UVC; a third of the sample was immediately frozen, a third was kept in water in the dark, and a third was kept in water and irradiated with UVA light. Dark repair (no photoreactivation) was very similar after 8 or 14 h of inoculation (Fig. 5A). The contribution of UVA to UV damage repair was stronger in the 8 h postinoculation than in the 14 h postinoculation group. During the first 2 h of photoreactivation, the repair was twice as efficient when cells were treated at 8 h postinoculation compared to that at 14 h postinoculation (Fig. 5B). We next determined the effect of irradiation timing and photoreactivation on the survival of cells irradiated with different UV doses. Without photoreactivation, there was a small effect of the timing of UVC irradiation on survival, whereas irradiated cells at 8 h postinoculation showed a higher survival rate. However, the major difference was observed under photoreactivation conditions. Photoreactivation improved the survival of irradiated cells at 8 h postinoculation (Fig. 5D). In contrast, for cells irradiated at 14 h postinoculation, survival with and without photoreactivation was almost the same (Fig. 5D). Taken together, the capacity to perform photoreactivation-dependent repair of UV lesions correlated with the pattern of *phr1* expression and with higher UV survival.

### An adaptation to living in the dark?

**FIG 6:**
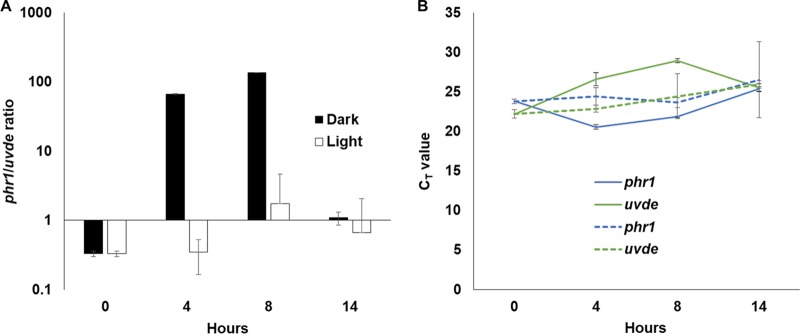
Short illumination period before inoculation abolishes the oscillations in *phr1* and *uvde* expression. Conidia were harvested in total darkness and then illuminated with fluorescent light for 10 min. The conidia were inoculated in PDB for 4, 8, and 14 h. RNA was harvested, and the expression of *phr1* and *uvde* was determined by quantitative PCR. (A) The ratio of *phr1* to *uvde* expression (as expressed by the inverse ratio of their quantitative PCR *C_T_* values) was determined at 0, 4, 8, and 14 h postinoculation. Light, 10 min of fluorescent illumination; dark, without illumination. (B) Quantitative PCR *C_T_* values of *phr1* and *uvde* at different time points postinoculation in untreated or illuminated conidia. The blue line represents *phr1*, while the green line represents *uvde*. Solid lines indicate dark, whereas dashed lines indicate 10 min of light illumination.

Fungi have a remarkable ability to respond to visible light. A short illumination period changes the expression of genes for hours afterward. We examined whether the periodic gene expression of *phr1* and *uvde* is changed by exposure to 10 min of a visible light prior to germination. Conidia were harvested in total darkness, exposed to fluorescent light for 10 min, and inoculated in PDB medium for either 4 or 8 h. Remarkably, under these conditions, no oscillations in the expression of *phr1* and *uvde* were observed (Fig. 6). We hypothesize that the oscillations in the expression of UV repair genes are an adaptation to living in the soil, in the dark, despite the fact that F. oxysporum retains the ability to respond to light.

## DISCUSSION

**FIG 7:**
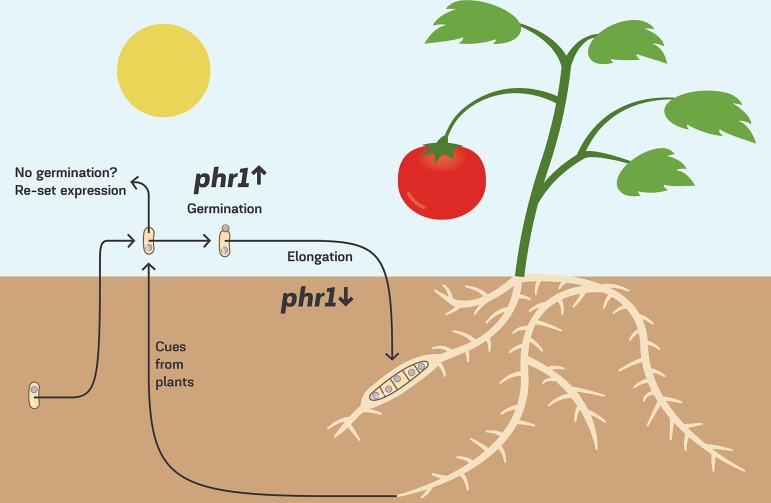
Transcription control of UV repair capacity during the life cycle of F. oxysporum. Shown is a model integrating the expression of UV repair genes in the soilborne fungal plant pathogen F. oxysporum with its life cycle and ecology.

The results described here were obtained with microconidia, the most common soilborne spores of F. oxysporum. As described earlier, F. oxysporum has two more types of spores. Chlamydospores are protected from UV by their melanized thick cell wall, but how germlings of chlamydospores react to UV is still unknown. Macroconidia are formed on the infected plants. Whether the results obtained here are relevant to macroconidia is interesting because they can be dispersed by wind (45). We propose a model to put our results in an ecological context. Soilborne microconidia of F. oxysporum f. sp. lycopersici are abruptly exposed to the sun near a tomato plant. Plant signals are perceived by the conidia and stimulate germination toward the tomato plants (13, 14) (Fig. 7). At this stage, exposure to the sun is risky for the survival and genome integrity of the germling, but due to a developmentally regulated program, the expression of *phr1* is high, and photoreactivation repair is strong (Fig. 3 and 5). When the germlings further develop, they penetrate the plant tissue. At this stage, the young hyphae are no longer exposed to UV damage on one hand but are also not exposed to visible light on the other hand. Therefore, unrepaired UV lesions cannot be repaired by photolyase. However, at this stage, the expression of *uvde* is upregulated, and dark repair can occur (Fig. 3, 5, and 7). In nongerminating microconidia that are exposed to the sun, the transcriptional program is changed to a plan that we think fits better an adaptation to light (Fig. 6). This adaptation of F. oxysporum to light yet has to be determined in higher resolution than what is described here (Text S3).

We do not know if the developmentally regulated oscillations in the expression of *uvde* and *phr1* are common to other soilborne fungi. The expression of *phr1* in the soilborne fungus Trichoderma harzianum has been shown to be developmentally regulated, because different amounts of the transcript are found in conidiophores, germinating conidia, and mature hyphae (39). However, *phr1* expression in T. harzianum was not examined through germination at high resolution, and the same trend may therefore hold in that fungus.

The observed oscillations in gene expression were dependent on cell cycle progression (Fig. 3 and 4). It has been previously shown that developmental stages in fungal plant pathogens are dependent on cell cycle progression. Appressorium formation and host penetration in the rice fungal pathogen Magnaporthe oryzae have been shown to depend on cell cycle progression and, specifically, completion of DNA replication (46). Similarly, in the barley fungal pathogen Blumeria graminis, appressorium morphology is disrupted by HU (47). Interestingly, a connection between the cell cycle, and in particular, DNA replication, and the circadian clock has been previously established in fungi (48, 49). It is possible that inhibition of the cell cycle resets the circadian clock, thus preventing or suppressing the oscillations in *phr1* and *uvde* expression.

In conclusion, we provide novel insight into the regulation of UV repair genes in a soilborne fungus. The demonstrated expression pattern provides a first step in understanding how soilborne fungi can survive abrupt UV exposure while reducing the cost of continuous expression of UV repair genes while in the soil.

## MATERIALS AND METHODS

### Purification and growth of conidia of F. oxysporum f. sp. *lycopersici*.

The starting point of all the experiments described here is resting microconidia (asexual spores) of the fungus F. oxysporum f. sp. *lycopersici* (strain 4287). Throughout the manuscript, they are termed conidia. To generate conidia for each set of experiments, aliquots from a frozen stock were incubated in KNO_3_-based medium (1.36 g yeast nitrogen base, 24 g sucrose, and 100 mM KNO_3_ in 800 ml distilled water) on an orbital shaker (250 rpm) at 28°C for 5 days. Conidia were obtained by filtering the culture through a nylon cell strainer (mesh size, 40 μm; Corning, USA) and resuspended in sterile water usually to a concentration of 1 × 10^9^ to 3 × 10^9^ conidia/ml. This stock of conidia was kept at 4°C for no more than 16 h before the experiments were initiated. This stock also served as time 0 in our experiments. In all of the experiments described here, conidia were inoculated into potato dextrose broth (PDB; BD Sparks, USA) at 0.1 to 1 × 10^8^ conidia/ml. The conidia were incubated on an orbital shaker at 28°C for the indicated time points (as described in the Results section). During the incubation, conidia germinate into germlings that, with time, mature to young hyphae. Finally, the germlings are harvested for either DNA or RNA purification or treated as specified.

### UV irradiation and sunlight exposure experiments.

Germlings (8 and 14 h postinoculation) were obtained by filtering 10 ml of the cultures that contained 2 × 10^7^ conidia/ml at time 0. The germlings were irradiated with either 50 or 200 J/m^2^ UV light (254 nm, 15 W lamp; Osram, Germany); the incident fluence was measured with a radiometer (YK-35UV; Digital Instruments, USA). Irradiation was followed by 0, 30, or 60 min of incubation in PDB at 28°C for recovery. To evaluate the impact of sunlight, 14-h-postinoculation germlings were exposed to sunlight for 2 h at 12:00 on a sunny day in the summer in Israel (12 July 2017). Both following UV and sun exposures, the germlings were harvested, immediately flash frozen in liquid N_2_, and kept at –80°C until RNA extraction was carried out.

### RNA extraction.

Conidia were disrupted in a Minilys bead beater (Bertin Instruments, France) for 30 s at medium speed. Total RNA was prepared with the Qiagen (USA) RNeasy microkit and was treated on-column with RQ1 RNase-free DNase (Promega, USA) to remove additional residues of genomic DNA. The RNA quality was measured by Agilent 2200 TapeStation machine using an RNA kit (Agilent Technologies, USA). For quantification, 1 μl RNA was measured with a Qubit 2.0 fluorometer and RNA BR kit (Life Technologies, USA).

### RNA-seq library construction and next-generation sequencing.

cDNA libraries were prepared using 0.5 μg RNA with a QuantSeq 3ʹ mRNA-seq library prep kit FWD (Lexogen GmbH, Austria) and were sequenced using the NextSeq 500 system (Illumina, USA) at the Center for Genomic Technologies of the Hebrew University of Jerusalem. Three biological replicates of each treatment were assessed.

### Transcriptome analysis.

Raw reads were mapped against the F. oxysporum 4287 (50) reference genome and were counted with HTSeq (51). The reads were assembled to genes, and the gene expression was normalized and quantified using the DESeq2 R package data normalization method (52). Differential expression was assessed for genes expressed in all combinations of UVC treatments versus untreated controls. DESeq2 was used to identify differentially expressed genes based on the negative binomial distribution, adjusted *P* values (*P < *0.05), and log_2_ fold change values of ≥+1 and ≤−1.

### Hierarchical clustering analysis.

Heat maps were generated using normalized read count values and the heatmap.2 function of the “gplot” R package (https://www.r-project.org).

### Analysis of Gene Ontology terms.

Functional enrichment analysis was performed using g:Profiler and one-tailed Fisher’s exact test. The g:SCS method was used to compute multiple testing correction with a *P *value of *≤*0.05 as the significance threshold (https://biit.cs.ut.ee/gprofiler/gost).

### cDNA synthesis and quantitative PCR expression analysis.

The RNA that was purified (see “RNA extraction,” above) was also used in a qPCR analysis to determine accurately the relative expression of target genes. cDNA was generated with a FastQuant reverse transcriptase (RT) kit (Tiangen, China), according to the manufacturer’s instructions, using 1 μg RNA in a 20-μl reaction mixture at 42°C. Quantitative PCR was carried out using Fast SYBR green master mix (Thermo Fisher Scientific). Each well contained 2 ng cDNA. The final reaction volume in each well was 10 μl in 0.1-ml 96-well plates (USA Scientific, USA). The reactions were carried out on a StepOnePlus real-time PCR system (Thermo Fisher Scientific). Data were analyzed using the StepOnePlus v2.2.3 software (Applied Biosystems). All data were compared using the comparative ΔΔ*C_T_* method (53). All primer pairs (see Table S1 for a complete list of primers used for all qPCR analyses) amplified their target with equal efficiencies (data not shown).

10.1128/mBio.02623-19.5 TABLE S1 Primers used in this study. Download Table S1, XLSX file, 0.1 MB. Copyright © 2019 Milo-Cochavi et al. 2019 Milo-Cochavi et al. This content is distributed under the terms of the Creative Commons Attribution 4.0 International license.

### Cell cycle arrest at different developmental stages.

To evaluate the impact of different developmental stages and cell cycle progression on the expression levels of UV repair genes, suspensions of 2 × 10^7^ conidia/ml were subjected to one of two different treatments, as follows: (i) incubation in either 10 ml PDB plus 200 mM hydroxyurea (HU) (Acros; Thermo Fisher Scientific, USA) or 10 μg/ml benomyl (Sigma, Israel) for 8 h, or (ii) incubation in 10 ml PDB for 8 h, followed by the addition of HU or benomyl, as described above, for another 6 h, for a total of 14 h of incubation. Incubations were performed on an orbital shaker (250 rpm) at 28°C. Germlings were harvested, immediately flash frozen in liquid N_2_, and kept at –80°C, followed by RNA purification and qPCR analysis.

To monitor the number of nuclei per conidia/germling, an F. oxysporum strain that encoded H1-GFP fusion at the native locus was used (44) (kindly provided by Antonio Di Pietro’s lab at the University of Córdoba). Images of conidia inoculated in PDB either with or without 200 mM or 50 μg/ml benomyl were taken at 0, 4, 8, and 14 h postinoculation using an SP8 microscope (Leica Microsystems, Wetzlar, Germany). Images were taken using Z stack, laser at 488 nm, with PMT detector range of 497 nm to 550 nm. The objective was HC PL APO CS2 40×/1.10 (water was used for immersion). The refraction index was 1.33 and zoom was 0.75. Statistical significance was determined by applying Pearson’s chi-square test for count data using the chi-square.test function of the “MASS” R package (https://www.r-project.org/).

### Survival assays.

For controlled-environment survival assays, 500 germlings (8 or 14 h postinoculation) were plated on potato dextrose agar (PDA; BD Sparks) medium and were subjected to acute UV (254 nm) treatments (50, 100, 150, and 200 J/m^2^). Half of the plates were immediately exposed to a UVA white light lamp (15 W; Philips, the Netherlands) at a distance of 25 cm at room temperature for 1 h. After 48 h of incubation at 28°C in total darkness, the surviving colonies were counted.

### Measuring repair efficiency of CPDs.

Filtered 8- and 14-h germlings were used in 10-ml suspensions of 2 × 10^7^ conidia/ml. Suspensions were irradiated with 50 J/m^2^ UV light (254 nm); then, half of the samples were subjected to photoreactivation, where spores were exposed to UVA light for 1, 2, 4, 6, and 8 h at a distance of 25 cm at room temperature. Spores were then harvested for analysis of DNA lesion formation and repair, immediately flash frozen in liquid N_2_, and kept at –80°C.

### Immunodot blot assay.

Irradiated conidia were disrupted using a Minilys bead beater for 60 s at medium speed. Genomic DNA was purified using 2% (wt/vol) hexadecyltrimethylammonium bromide (CTAB) buffer, followed by a chloroform-isoamyl alcohol (24:1 [vol/vol]) phase separation procedure and precipitation in a final concentration of 50% ice-cold isopropanol. An immunodot blot assay was used to quantify DNA lesions, as described previously (54). Briefly, after denaturation at 95°C for 10 min, 200 ng DNA was combined with an equal volume of 2 M ammonium acetate and placed on ice. Each DNA sample was spotted onto a nitrocellulose membrane (soaked in 6× saline sodium citrate buffer for 10 min at room temperature) in duplicate, using a Minifold dot blot manifold (Schleicher & Schuell, the Netherlands). Membranes were dried in a vacuum gel dryer (model 583; Bio-Rad, USA) for 90 min at 80°C. After blocking in 5% powdered milk in PBST (1 ml Tween 20, 100 ml of 10× phosphate-buffered saline [Biological Industries, Israel], 899 ml double-distilled water), membranes were probed with mouse anti-CPD antibody (NMDND001; Cosmo-Bio, Japan). Following secondary antibody application (peroxidase-conjugated; Jackson ImmunoResearch Laboratories, USA), enhanced chemiluminescence was used to detect the antibody-dependent signal from each DNA spot on-film. The intensity of each spot was quantified using the ImageJ software.

### Pretreatment with visible light before inoculation.

To evaluate the influence of light exposure before the initiation of conidial germination on the expression levels of UV repair genes at different developmental stages, conidia were obtained by filtering the cultures in total darkness, and suspensions of 10 ml with 2 × 10^7^ conidia/ml were illuminated with fluorescent light (Philips 32-W lamp) for 10 min. Illumination was followed by either 4, 8, or 14 h of incubation in PDB on an orbital shaker at 28°C in total darkness. Germlings were harvested in total darkness, immediately flash frozen in liquid N_2_, and kept at –80°C, followed by RNA purification and qPCR analysis.

10.1128/mBio.02623-19.1 TEXT S1 Global analysis of the response to UV radiation in germinating conidia of F. oxysporum. Download Text S1, PDF file, 0.5 MB. Copyright © 2019 Milo-Cochavi et al. 2019 Milo-Cochavi et al. This content is distributed under the terms of the Creative Commons Attribution 4.0 International license.

10.1128/mBio.02623-19.2 TEXT S2 Analysis of the response of UV repair genes to UV radiation in germinating conidia of F. oxysporum. Download Text S2, PDF file, 0.1 MB. Copyright © 2019 Milo-Cochavi et al. 2019 Milo-Cochavi et al. This content is distributed under the terms of the Creative Commons Attribution 4.0 International license.

10.1128/mBio.02623-19.3 TEXT S3 Analysis of the response of UV repair and circadian clock genes to sunlight in germinating conidia of F. oxysporum. Download Text S3, PDF file, 0.1 MB. Copyright © 2019 Milo-Cochavi et al. 2019 Milo-Cochavi et al. This content is distributed under the terms of the Creative Commons Attribution 4.0 International license.

10.1128/mBio.02623-19.4 TEXT S4 NER gene and MMS damage repair gene expression does not oscillate during the germination of F. oxysporum. Download Text S4, PDF file, 0.1 MB. Copyright © 2019 Milo-Cochavi et al. 2019 Milo-Cochavi et al. This content is distributed under the terms of the Creative Commons Attribution 4.0 International license.
