# Supplementary material for: Developmentally Regulated Oscillations in the Expression of UV Repair Genes in a Soilborne Plant Pathogen Dictate UV Repair Efficiency and Survival
Source: mBio. 2019 Dec 3;10(6):e02623-19. doi: 10.1128/mBio.02623-19 (PMC6890992; doi:10.1128/mBio.02623-19)

Global Analysis of the Response to UV Radiation in Germinating Conidia of *Fusarium oxysporum*

Experimental Procedures

250 – 500 million conidia were grown in PDB for 8 or 14 h at 28 °C 250 rpm. Then, cells were filtered, centrifuged at 4000 rpm 4 °C for 10 min, and re-suspended in iced cold DDW. Non-irradiated samples were kept at 4 degree. Irradiation was done as described in Materials and Methods. After irradiation cells were centrifuged again and re-suspended in a warm PDB medium (including non-irradiated samples). The samples were incubated in 28 °C 250 rpm for 0, 30 and 60 min for recovery (non-irradiated samples were harvested right after re-suspension). RNA was purified as described in Materials and Methods. RNA library preparation was according to the protocol of 3' quantseq of Lexogen. Only the 3' ends of mRNA are sequenced in this protocol. RNA sequencing and analysis were done as described in Material and Methods. Functional enrichment analysis was performed using g:Profiler and one-tailed Fisher's exact test. The g:SCS method was used to compute multiple testing correction with $P \leq 0.05$ as the significance threshold (http://biit.cs.ut.ee/gprofiler).

Results

Conidia that were irradiated 14 h postinoculation showed an increase in the transcription of translation-related genes, mainly components of the ribosome, processes of ribosome biogenesis such as rRNA processing. This is true even when there is no recovery time – indicating that the change in gene expression is very fast. Only one informative GO term was down-regulated - oxireductases. Interestingly, when the cultures were irradiated 8 h postinoculation the down-regulated GO terms were very similar to the ones that were up-regulated when cells were irradiated 14 h postinoculation (translation related genes). As described in detail in our result section, we think this is due to the basal level of expression at different time points postinoculation. The GO terms of up-regulated genes when cell were irradiated with 50 J/m^2 8 h postinoculation were general and not informative. GO terms of up-regulated genes when 8 h postinoculation cells were irradiated with 200 J/m^2 were acetyltransferase activity. There are several histone acetyltransferase genes that are up-regulated. Further investigation is needed to understand the role of these histone acetyltransferases in the response to the UV damage response. For all graphs below the Y axis is 1/P value and the X axis is GO terms

UV irradiation 14 h postinoculation

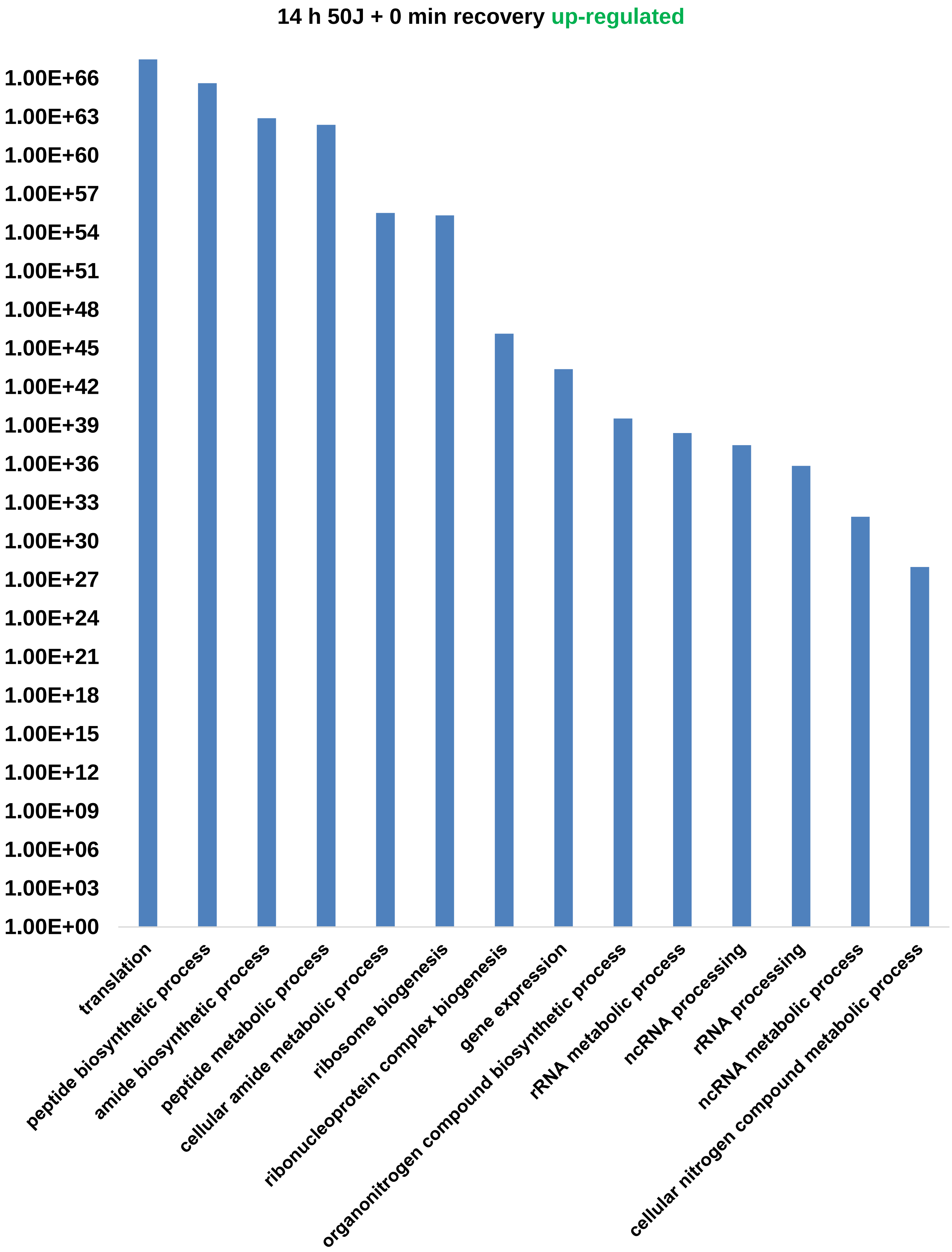

14 h 50J + 0 min recovery up-regulated
1.00E+66
1.00E+63
1.00E+60
1.00E+57
1.00E+54
1.00E+51
1.00E+48
1.00E+45
1.00E+42
1.00E+39
1.00E+36
1.00E+33
1.00E+30
1.00E+27
1.00E+24
1.00E+21
1.00E+18
1.00E+15
1.00E+12
1.00E+09
1.00E+06
1.00E+03
1.00E+00
translation
peptide biosynthetic process
amide biosynthetic process
peptide metabolic process
cellular amide metabolic process
ribosome biogenesis
ribonucleoprotein complex biogenesis
gene expression
organonitrogen compound biosynthetic process
rRNA metabolic process
ncRNA processing
rRNA processing
ncRNA metabolic process
cellular nitrogen compound metabolic process

14 h 50J + 30 min recovery up-regulated

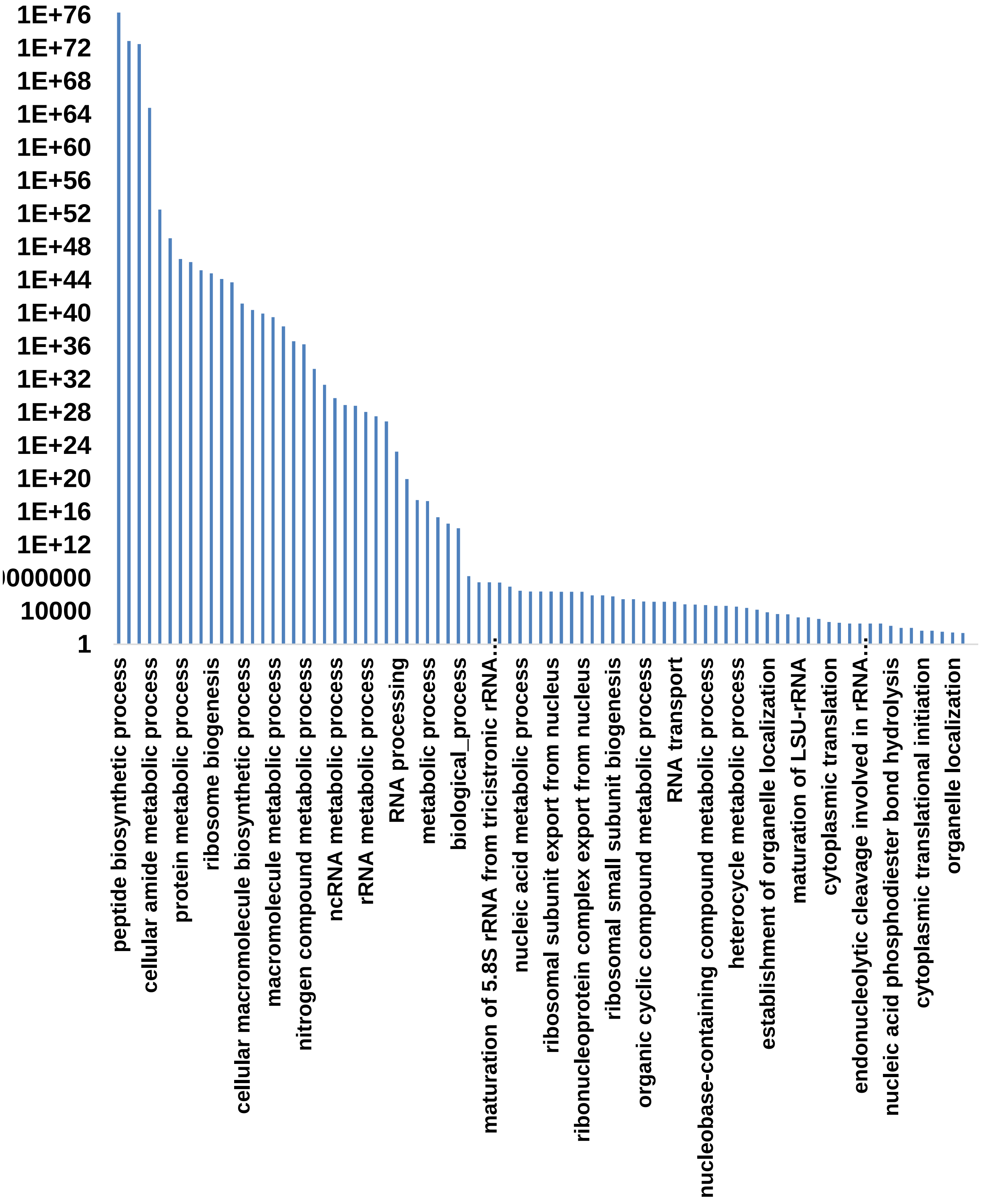

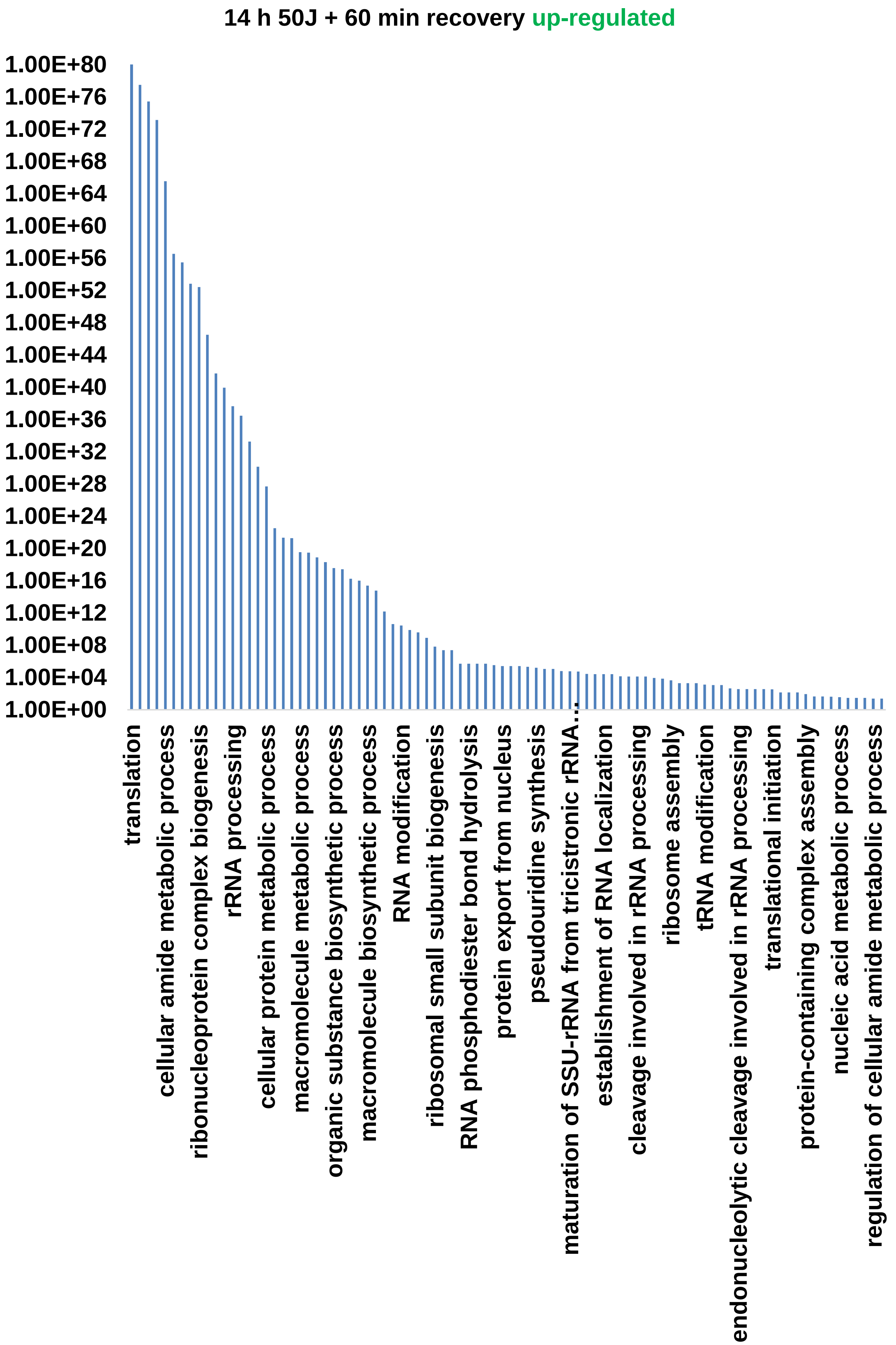
14 h 50J + 60 min recovery up-regulated
1.00E+80
1.00E+76
1.00E+72
1.00E+68
1.00E+64
1.00E+60
1.00E+56
1.00E+52
1.00E+48
1.00E+44
1.00E+40
1.00E+36
1.00E+32
1.00E+28
1.00E+24
1.00E+20
1.00E+16
1.00E+12
1.00E+08
1.00E+04
1.00E+00
translation
cellular amide metabolic process
ribonucleoprotein complex biogenesis
rRNA processing
cellular protein metabolic process
macromolecule metabolic process
organic substance biosynthetic process
macromolecule biosynthetic process
RNA modification
ribosomal small subunit biogenesis
RNA phosphodiester bond hydrolysis
protein export from nucleus
pseudouridine synthesis
maturation of SSU-rRNA from tricistronic rRNA…
establishment of RNA localization
cleavage involved in rRNA processing
ribosome assembly
tRNA modification
endonucleolytic cleavage involved in rRNA processing
translational initiation
protein-containing complex assembly
nucleic acid metabolic process
regulation of cellular amide metabolic process

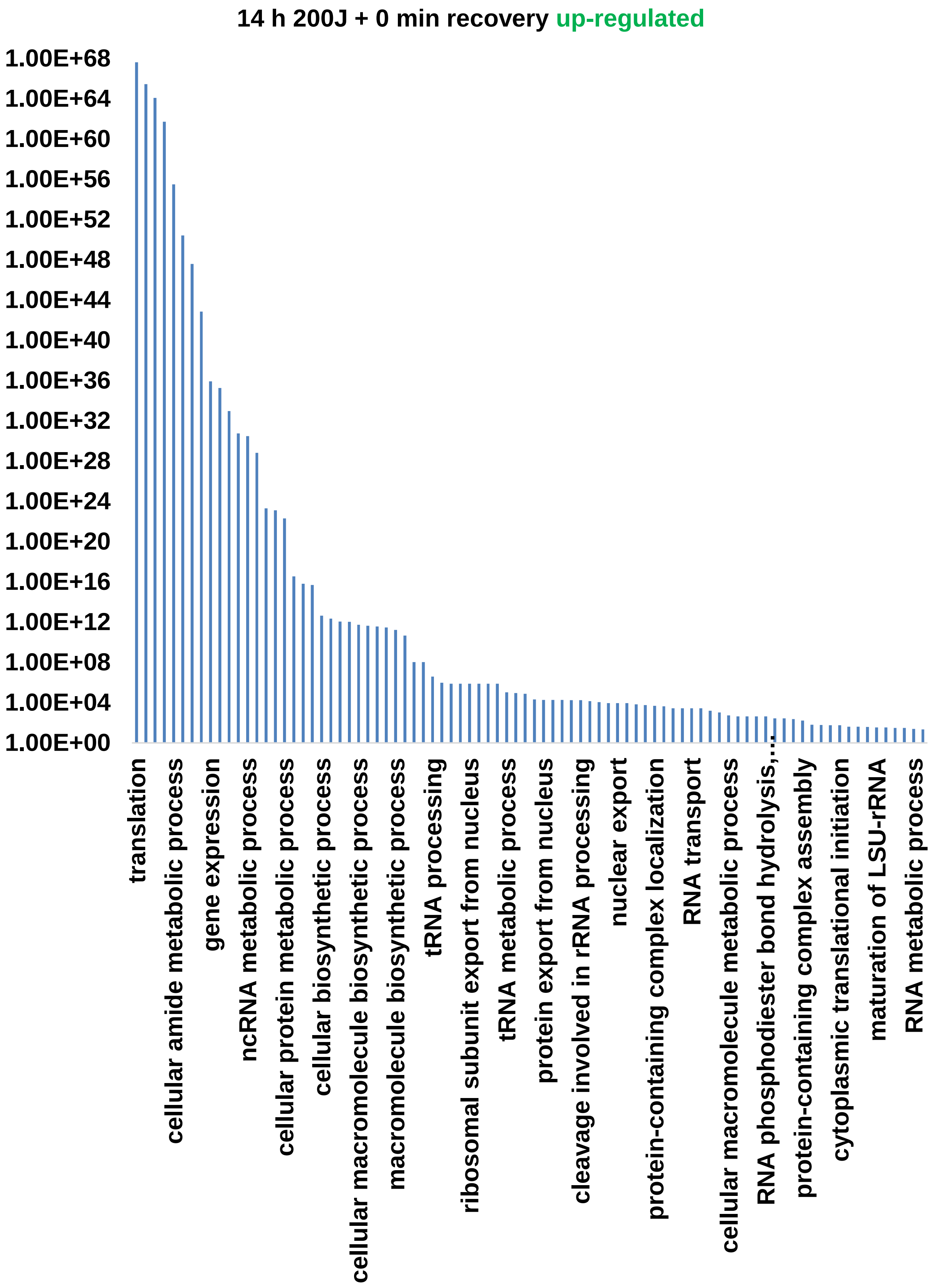

14 h 200J + 0 min recovery up-regulated
1.00E+68
1.00E+64
1.00E+60
1.00E+56
1.00E+52
1.00E+48
1.00E+44
1.00E+40
1.00E+36
1.00E+32
1.00E+28
1.00E+24
1.00E+20
1.00E+16
1.00E+12
1.00E+08
1.00E+04
1.00E+00
translation
cellular amide metabolic process
gene expression
ncRNA metabolic process
cellular protein metabolic process
cellular biosynthetic process
cellular macromolecule biosynthetic process
macromolecule biosynthetic process
tRNA processing
ribosomal subunit export from nucleus
tRNA metabolic process
protein export from nucleus
cleavage involved in rRNA processing
nuclear export
protein-containing complex localization
RNA transport
cellular macromolecule metabolic process
RNA phosphodiester bond hydrolysis,...
protein-containing complex assembly
cytoplasmic translational initiation
maturation of LSU-rRNA
RNA metabolic process

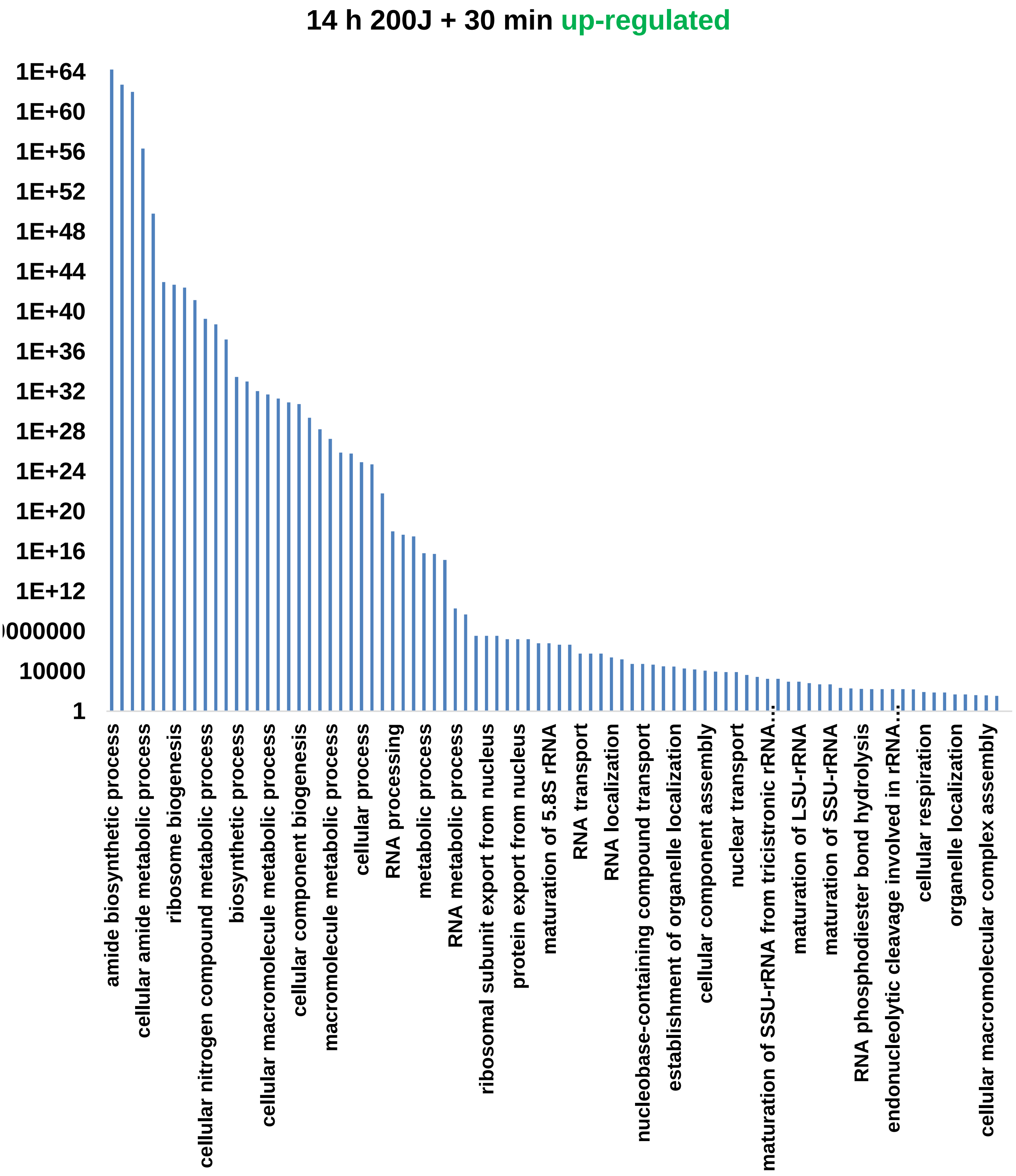
14 h 200J + 30 min up-regulated
1E+64
1E+60
1E+56
1E+52
1E+48
1E+44
1E+40
1E+36
1E+32
1E+28
1E+24
1E+20
1E+16
1E+12
000000
10000
1
amide biosynthetic process
cellular amide metabolic process
ribosome biogenesis
cellular nitrogen compound metabolic process
biosynthetic process
cellular macromolecule metabolic process
cellular component biogenesis
macromolecule metabolic process
cellular process
RNA processing
metabolic process
RNA metabolic process
ribosomal subunit export from nucleus
protein export from nucleus
maturation of 5.8S rRNA
RNA transport
RNA localization
nucleobase-containing compound transport
establishment of organelle localization
cellular component assembly
nuclear transport
maturation of SSU-rRNA from tricistronic rRNA…
maturation of LSU-rRNA
maturation of SSU-rRNA
RNA phosphodiester bond hydrolysis
endonucleolytic cleavage involved in rRNA…
cellular respiration
organelle localization
cellular macromolecular complex assembly

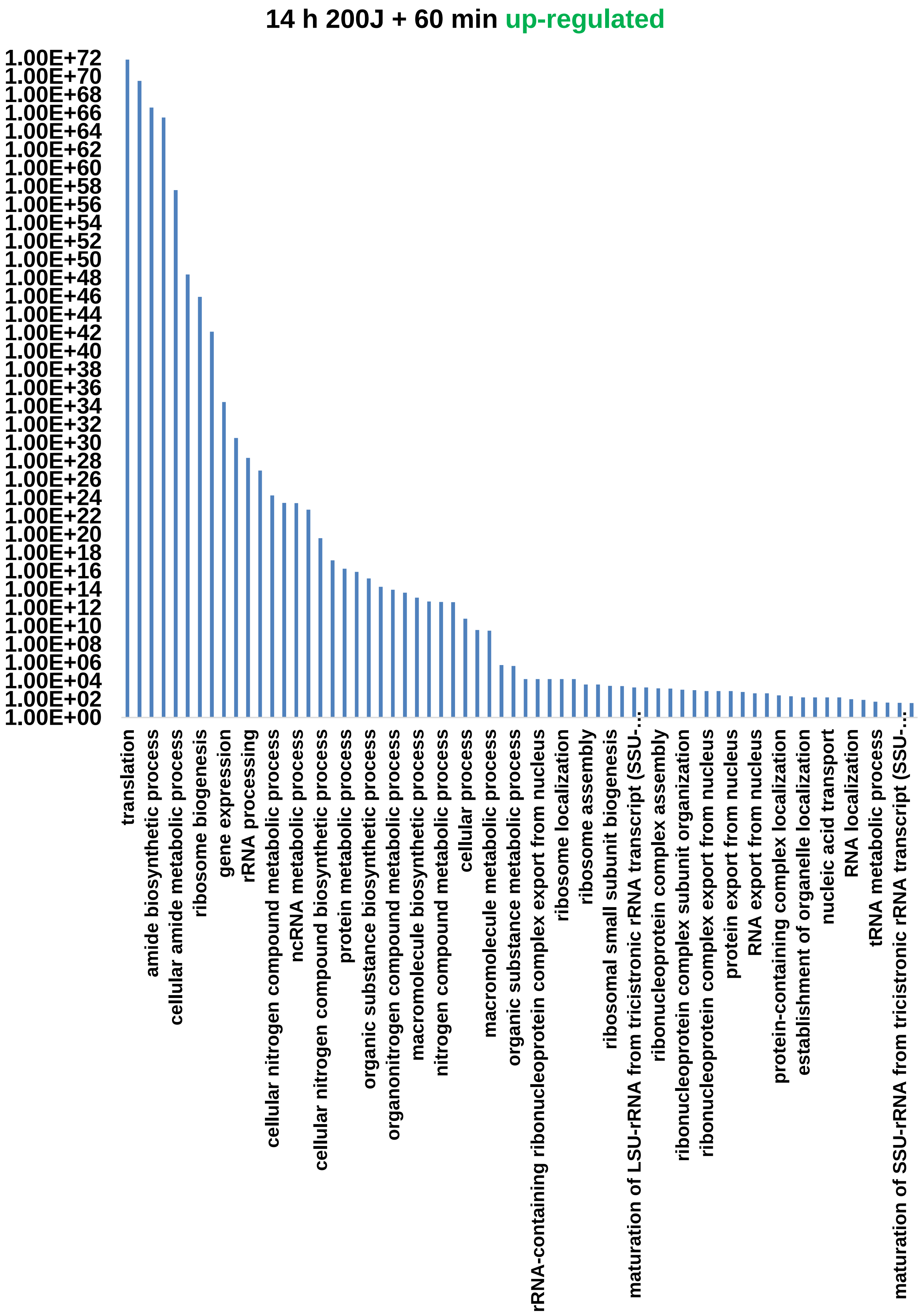
14 h 200J + 60 min up-regulated
1.00E+72
1.00E+70
1.00E+68
1.00E+66
1.00E+64
1.00E+62
1.00E+60
1.00E+58
1.00E+56
1.00E+54
1.00E+52
1.00E+50
1.00E+48
1.00E+46
1.00E+44
1.00E+42
1.00E+40
1.00E+38
1.00E+36
1.00E+34
1.00E+32
1.00E+30
1.00E+28
1.00E+26
1.00E+24
1.00E+22
1.00E+20
1.00E+18
1.00E+16
1.00E+14
1.00E+12
1.00E+10
1.00E+08
1.00E+06
1.00E+04
1.00E+02
1.00E+00
translation
amide biosynthetic process
cellular amide metabolic process
ribosome biogenesis
gene expression
rRNA processing
cellular nitrogen compound metabolic process
ncRNA metabolic process
cellular nitrogen compound biosynthetic process
protein metabolic process
organic substance biosynthetic process
organonitrogen compound metabolic process
macromolecule biosynthetic process
nitrogen compound metabolic process
cellular process
macromolecule metabolic process
organic substance metabolic process
rRNA-containing ribonucleoprotein complex export from nucleus
ribosome localization
ribosome assembly
ribosomal small subunit biogenesis
maturation of LSU-rRNA from tricistronic rRNA transcript (SSU-…
ribonucleoprotein complex assembly
ribonucleoprotein complex subunit organization
ribonucleoprotein complex export from nucleus
protein export from nucleus
RNA export from nucleus
protein-containing complex localization
establishment of organelle localization
nucleic acid transport
RNA localization
tRNA metabolic process
maturation of SSU-rRNA from tricistronic rRNA transcript (SSU-…

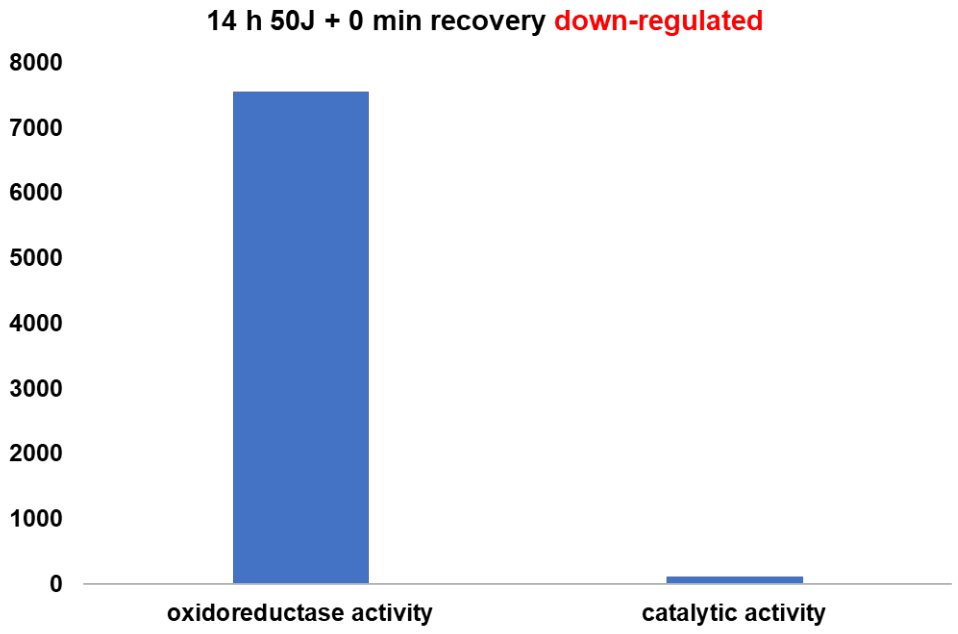
14 h 50J + 0 min recovery down-regulated
8000
7000
6000
5000
4000
3000
2000
1000
0
oxidoreductase activity
catalytic activity

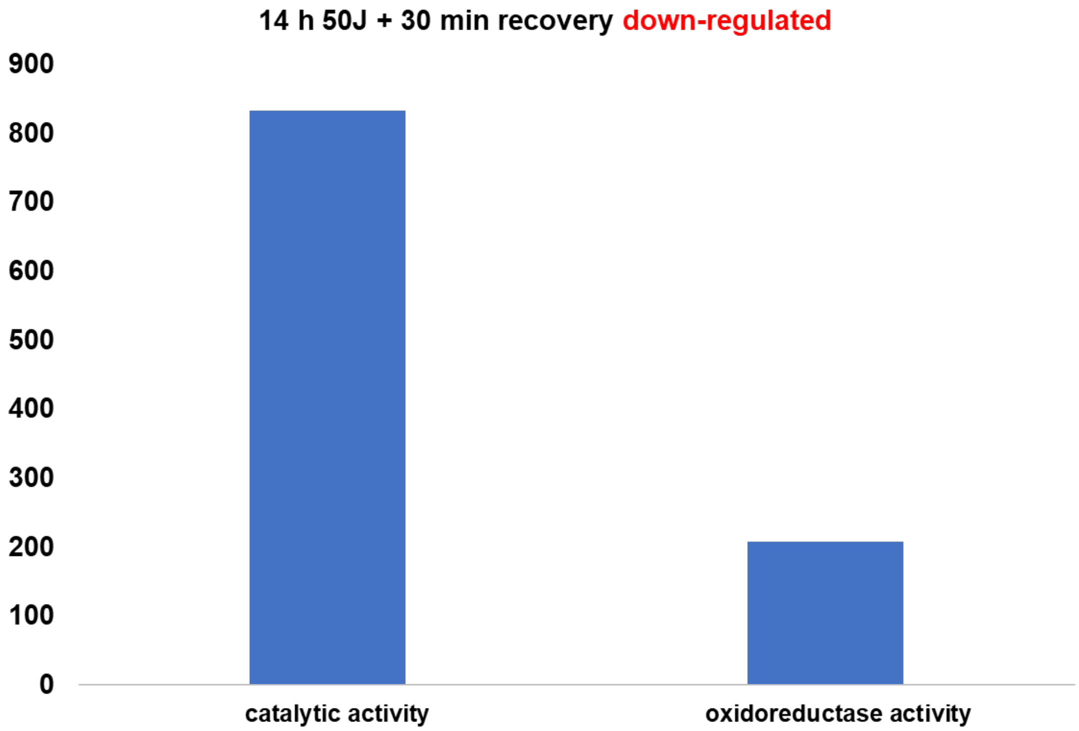
14 h 50J + 30 min recovery down-regulated
900
800
700
600
500
400
300
200
100
0
catalytic activity
oxidoreductase activity

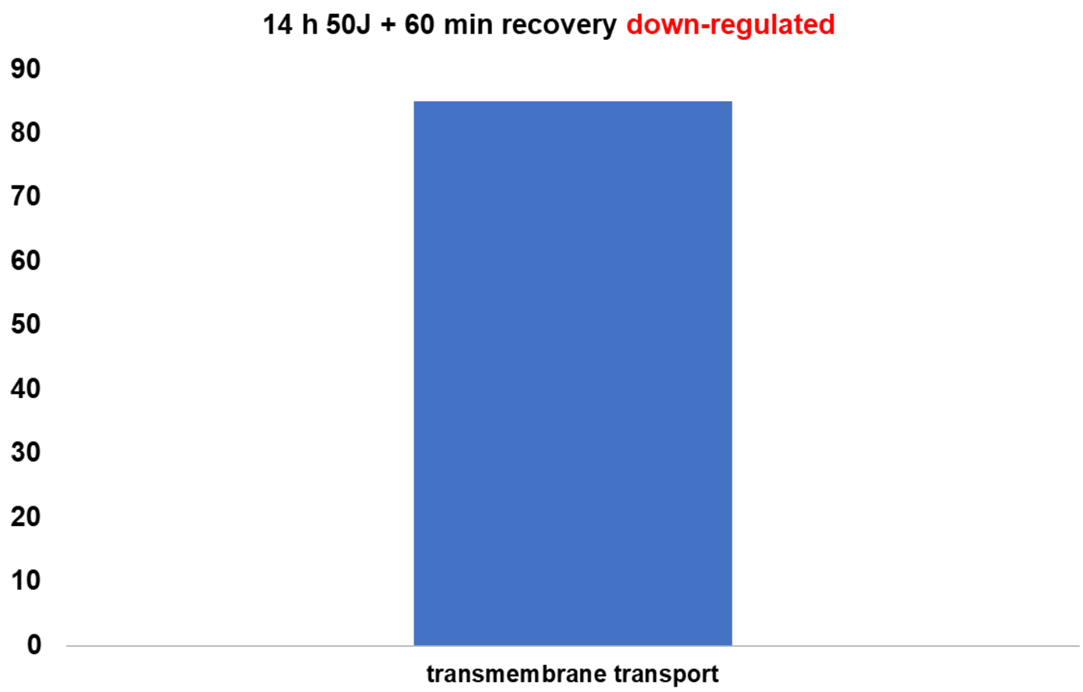
14 h 50J + 60 min recovery down-regulated
90
80
70
60
50
40
30
20
10
0
transmembrane transport

UV irradiation 8 h postinoculation

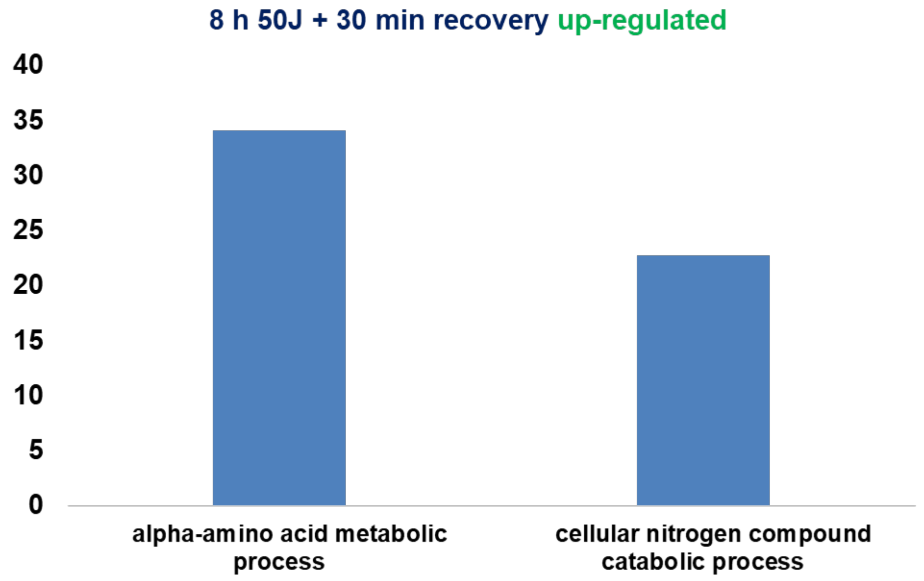

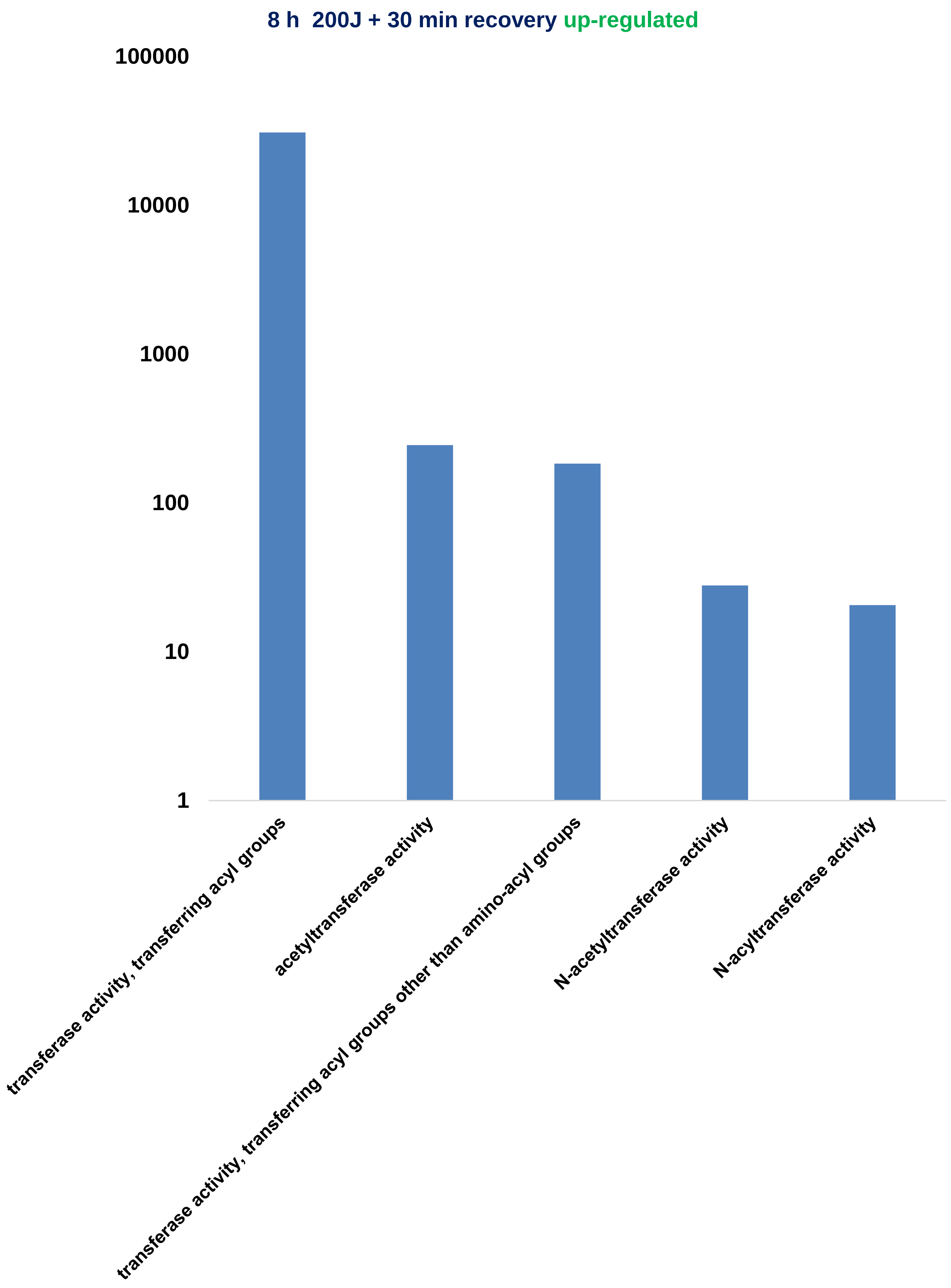
8 h 200J + 30 min recovery up-regulated
100000
10000
1000
100
10
1
transferase activity, transferring acyl groups
acetyltransferase activity
transferase activity, transferring acyl groups other than amino-acyl groups
N-acetyltransferase activity
N-acyltransferase activity

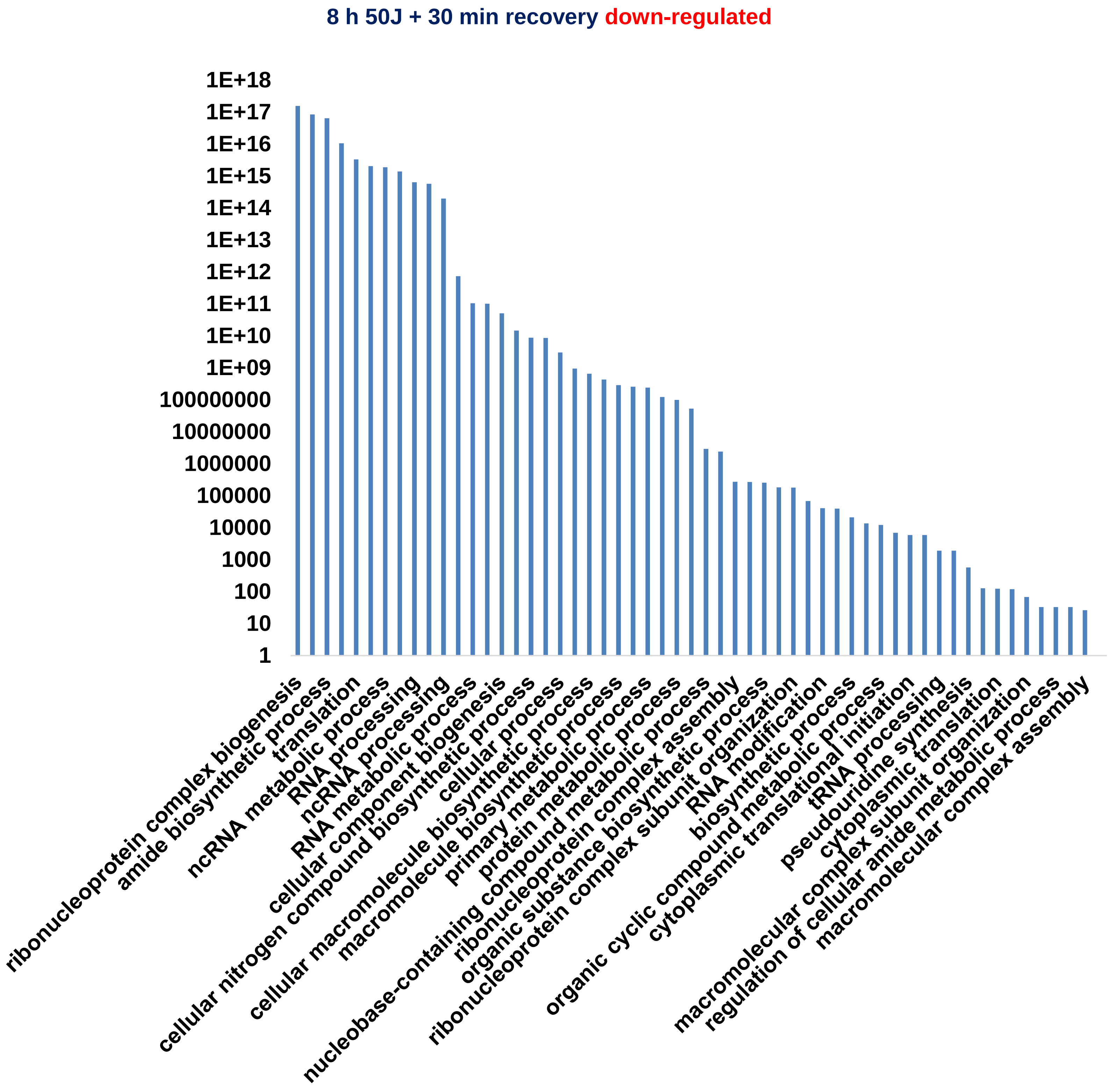
8 h 50J + 30 min recovery down-regulated
1E+18
1E+17
1E+16
1E+15
1E+14
1E+13
1E+12
1E+11
1E+10
1E+09
100000000
10000000
1000000
100000
10000
1000
100
10
1
ribonucleoprotein complex biogenesis
amide biosynthetic process
translation
ncRNA metabolic process
RNA processing
ncRNA processing
RNA metabolic process
cellular component biogenesis
cellular nitrogen compound biosynthetic process
cellular process
cellular macromolecule biosynthetic process
macromolecule biosynthetic process
primary metabolic process
protein metabolic process
nucleobase-containing compound metabolic process
ribonucleoprotein complex assembly
organic substance biosynthetic process
ribonucleoprotein complex subunit organization
RNA modification
biosynthetic process
organic cyclic compound metabolic process
cytoplasmic translational initiation
tRNA processing
pseudouridine synthesis
cytoplasmic translation
macromolecular complex subunit organization
regulation of cellular amide metabolic process
macromolecular complex assembly

8 h 200J + 30 min recovery down-regulated

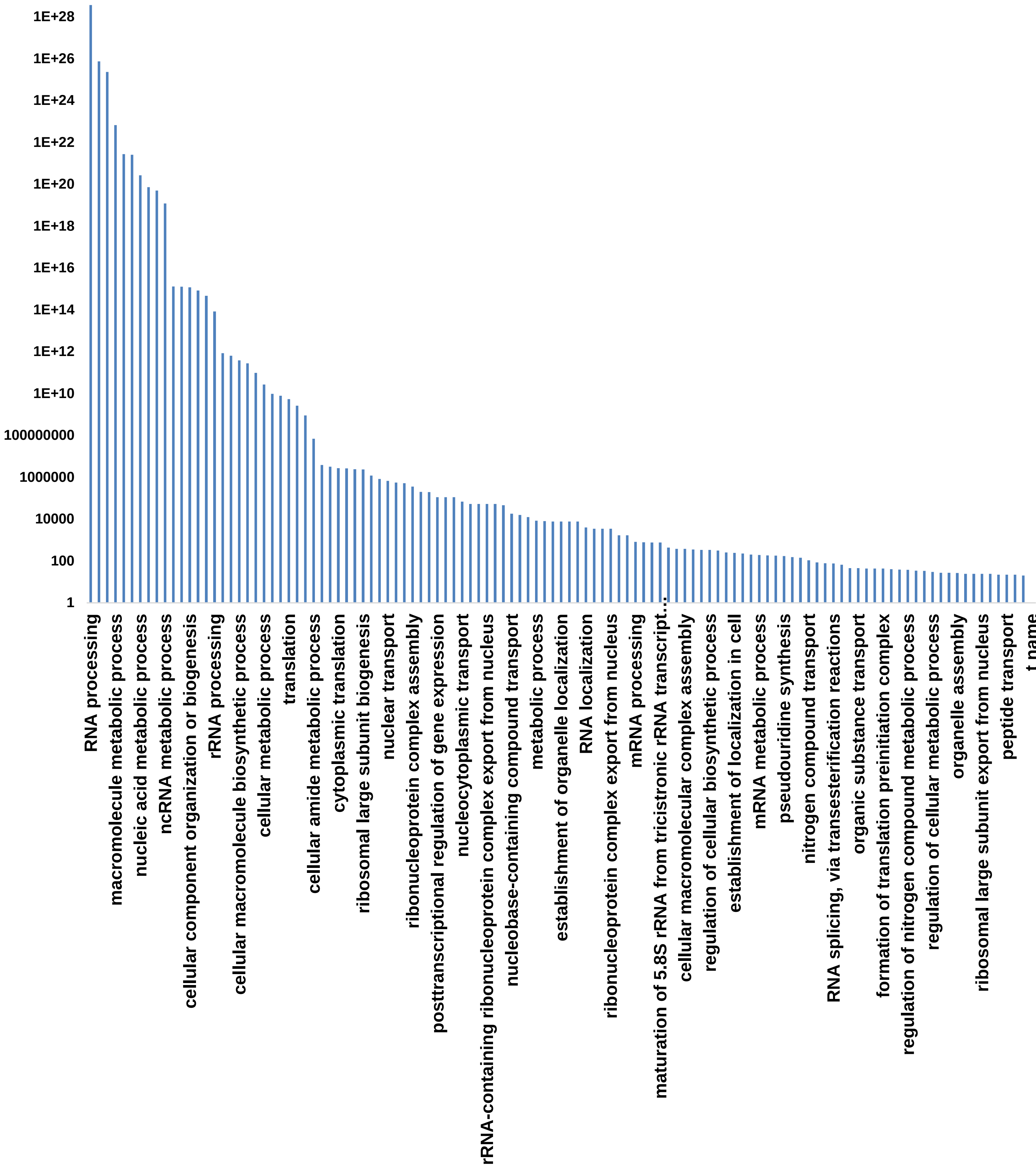

Supplement: TEXT S1 [file mBio.02623-19-s0001.pdf]
