# Supplementary material for: Developmentally Regulated Oscillations in the Expression of UV Repair Genes in a Soilborne Plant Pathogen Dictate UV Repair Efficiency and Survival
Source: mBio. 2019 Dec 3;10(6):e02623-19. doi: 10.1128/mBio.02623-19 (PMC6890992; doi:10.1128/mBio.02623-19)
Supplement: TEXT S2 [file mBio.02623-19-s0002.pdf]

Analysis of the Response of UV Repair Genes to UV Radiation in Germinating Conidia of *Fusarium oxysporum*

Results

The experimental procedures are as described in Supplementary File 1. The expression of 24/26 genes that are involved in DNA repair and DNA replication stress was compared between different UV exposures (No UV, 50 and 200 J/m^2) and different recovery times (0, 30 and 60 minutes). In the case of irradiation of 8 h postinoculation the data for 24 genes is presented because no reads were mapped to RAD30 and REV7. The results are presented as heatmaps that cluster genes according to the effect of UV treatments and recovery times. Heatmaps were generated using normalized read count values and the heatmap.2 function of the 'gplot' R package (https://www.r-project.org). Two developmental stages were analyzed; 14 h postinoculation (A) and 8 h postinoculation (B). At 14 h postinoculation the response to 200 J/m^2 resulted in down regulation of the expression of most genes, a trend that was stronger with incubation time. At this time point the effect of 50 J/m^2 was somewhat different, the expression some genes was induced and of other was reduced with no clear, common, functions and no clear trend of the recovery time. The analysis for irradiation 8 h postinoculation was not as detailed but showed clearly that several genes especially from the nucleotide excision repair pathway are induced by UV (RAD2, RAD4, RAD16 RAD28, XPC). It is noticeable that the effect of 50 J/m^2 is more profound than 200 J/m^2. Most of the genes described above are induced to higher level by 50 J/m^2 than 200 J/m^2. Only UVDE, MUS81 and RAD18 are induced by 200 J/m^2 more than 50 J/m^2. This could be due the stress high dose of UV imposes on transcription.

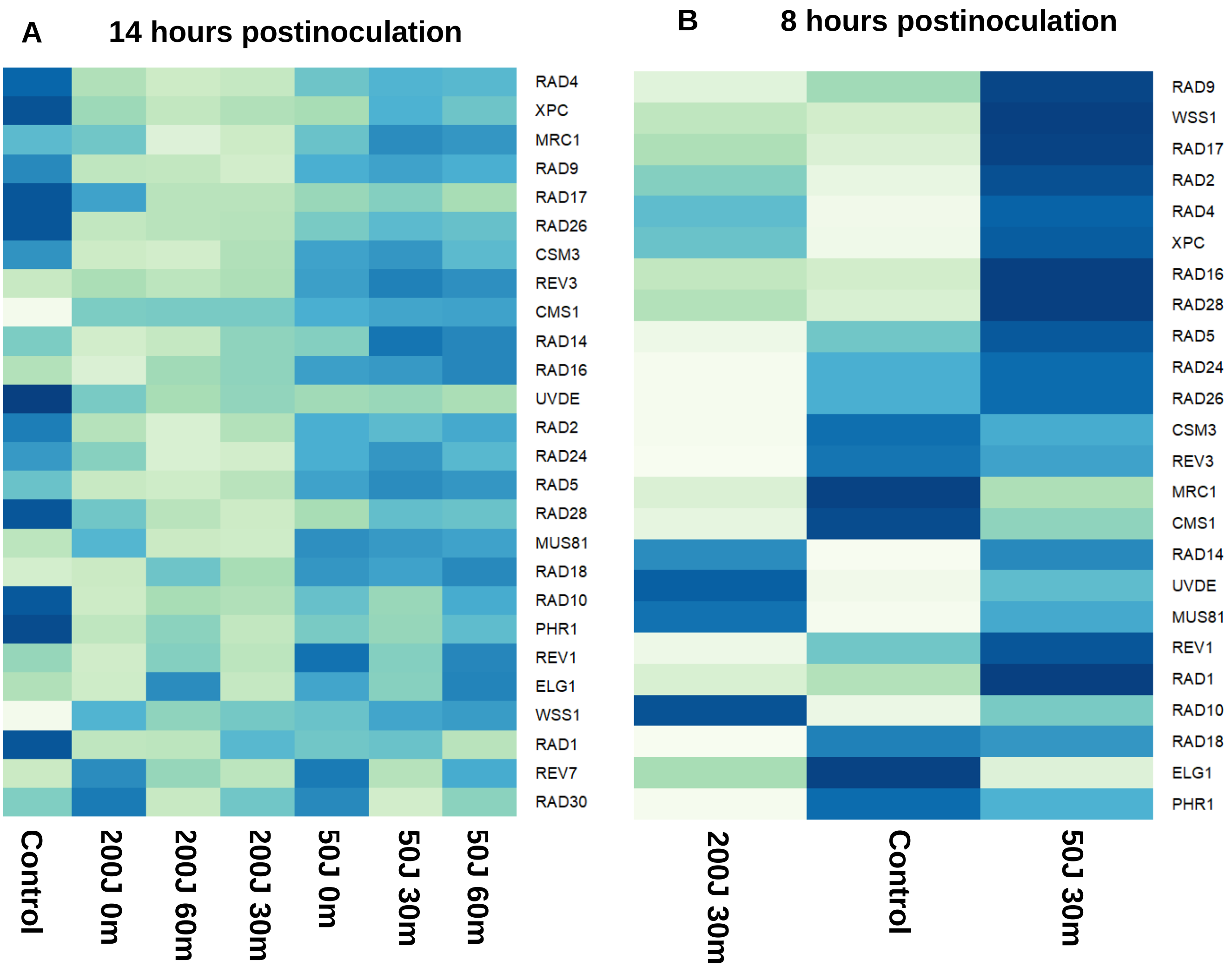
A
14 hours postinoculation
RAD4
XPC
MRC1
RAD9
RAD17
RAD26
CSM3
REV3
CMS1
RAD14
RAD16
UVDE
RAD2
RAD24
RAD5
RAD28
MUS81
RAD18
RAD10
PHR1
REV1
ELG1
WSS1
RAD1
REV7
RAD30
Control
200J 0m
200J 60m
200J 30m
50J 0m
50J 30m
50J 60m
B
8 hours postinoculation
RAD9
WSS1
RAD17
RAD2
RAD4
XPC
RAD16
RAD28
RAD5
RAD24
RAD26
CSM3
REV3
MRC1
CMS1
RAD14
UVDE
MUS81
REV1
RAD1
RAD10
RAD18
ELG1
PHR1
200J 30m
Control
50J 30m
