# Supplementary material for: Developmentally Regulated Oscillations in the Expression of UV Repair Genes in a Soilborne Plant Pathogen Dictate UV Repair Efficiency and Survival
Source: mBio. 2019 Dec 3;10(6):e02623-19. doi: 10.1128/mBio.02623-19 (PMC6890992; doi:10.1128/mBio.02623-19)
Supplement: TEXT S3 [file mBio.02623-19-s0003.pdf]

Analysis of the Response of UV Repair and Circadian Clock Genes to Sunlight in Germinating Conidia of *Fusarium oxysporum*

Experimental procedures

250 million conidia were grown in PDB for 14 or 8 h at 28 °C 250 rpm. Then, cells were filtered, centrifuged at 4000 rpm 4 °C for 10 min, and re-suspended in DDW. Non-exposed (control) samples were kept at room temperature. Sun exposure was done by placing petri dishes containing 10 ml conidia suspension on a table in bright sun for two hours with frequent swirling of the plates. After exposure cells were frozen. RNA purification, preparation of sequencing libraries and functional enrichment analysis was done as described in Materials and Methods and in Supplementary File 1.

Results

Only one GO term was significantly enriched following sun exposure – response to oxidative stress (A). The expression of 26 DNA repair genes that are involved in DNA repair and DNA replication stress as well as the expression of circadian clock genes (marked with *) were compared between exposed and control samples. The results are presented as a heat map that clusters genes according to the effect of sun exposure (B). Overall the effect of sun exposure on these genes was mild.

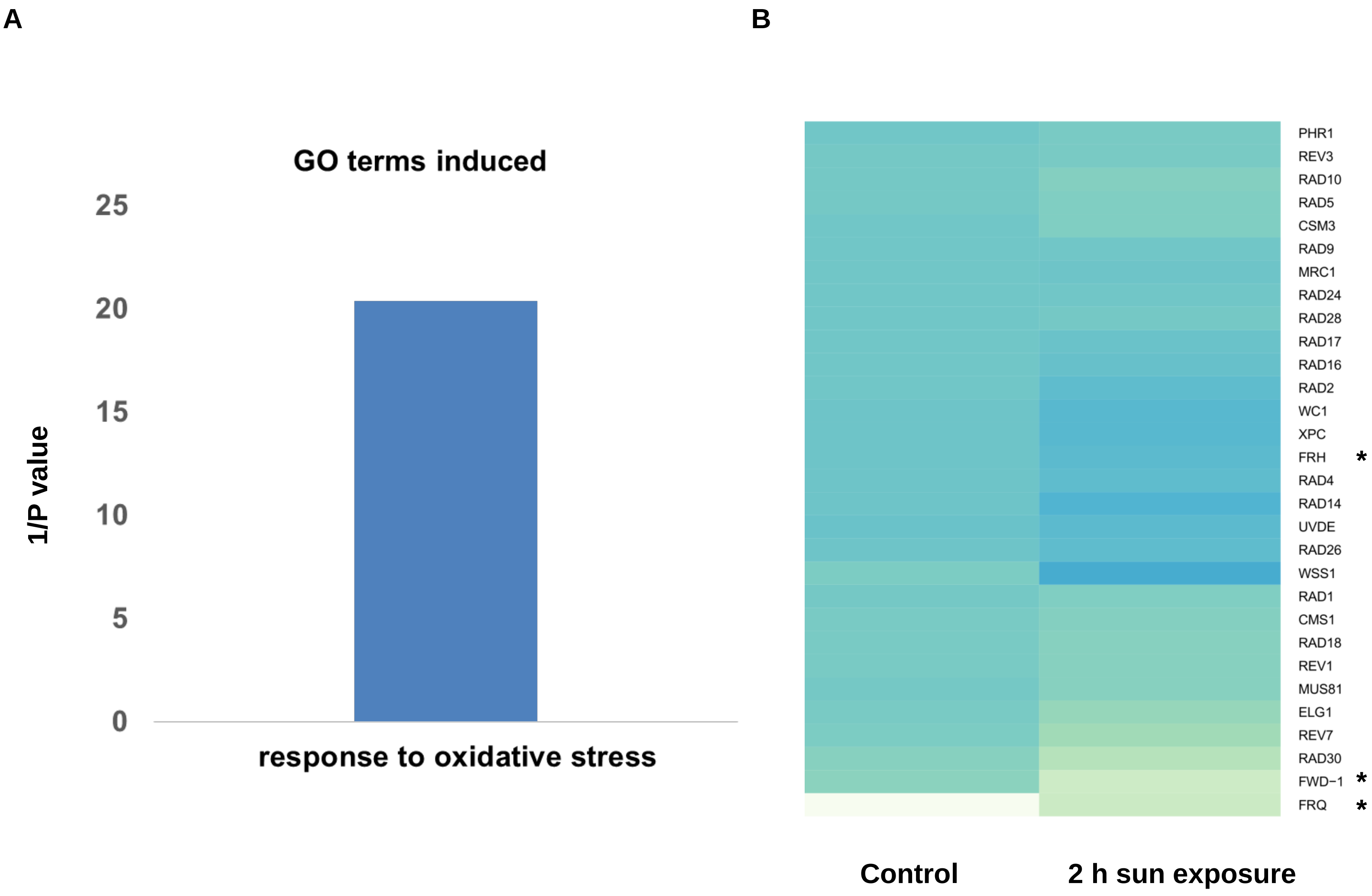
A
GO terms induced
25
20
15
10
5
0
1/P value
response to oxidative stress
B
PHR1
REV3
RAD10
RAD5
CSM3
RAD9
MRC1
RAD24
RAD28
RAD17
RAD16
RAD2
WC1
XPC
FRH *
RAD4
RAD14
UVDE
RAD26
WSS1
RAD1
CMS1
RAD18
REV1
MUS81
ELG1
REV7
RAD30
FWD−1 *
FRQ *
Control
2 h sun exposure
