# Supplementary material for: Developmentally Regulated Oscillations in the Expression of UV Repair Genes in a Soilborne Plant Pathogen Dictate UV Repair Efficiency and Survival
Source: mBio. 2019 Dec 3;10(6):e02623-19. doi: 10.1128/mBio.02623-19 (PMC6890992; doi:10.1128/mBio.02623-19)
Supplement: TEXT S4 [file mBio.02623-19-s0004.pdf]

NER Genes and MMS Damage Repair Genes Do Not Oscillate During the Germination of *F. oxysporum*

The levels of expression of DNA repair genes during germination of *F. oxysporum* conidia are presented as C_T values of the qPCR reactions (the time presented in X axis is hours post inoculation). (A) UV-specific repair genes. (B) Nucleotide excision repair genes. (C) Zoom-in to two NER genes, *ddb1* and *xpc* that show some oscillation in their expression. (D) Genes involved in repair of MMS-associated lesions. *mag1* is a lesion-specific glycosylase that participates in basic excision repair. *mgt1* is a methyltransferase, similar to *phr1*, it is specific to a lesion and its biochemical activity reverses the damaged base to the intact form without removing it. The results show that the oscillations of the expression of *uvde* and *phr1* are not common among DNA repair genes whether they belong to NER, a general repair mechanism, or *mag1* and *mgt1* that are lesion specific. The results presented in panels B and D show that the oscillations of the C_T values of *uvde* and *phr1* are not due to difference in RNA quantity, quality or cDNA convergence efficiency in the different time points after inoculations.

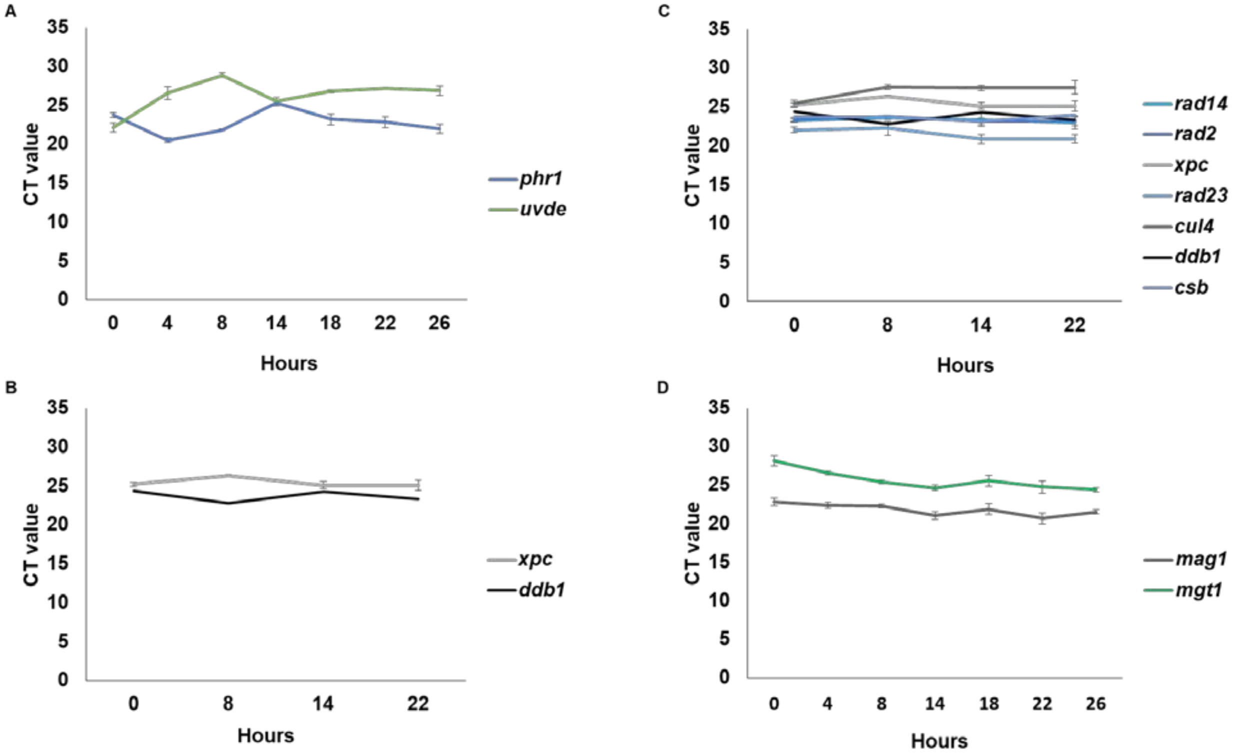
A
CT value
Hours
0 4 8 14 18 22 26
0 5 10 15 20 25 30 35
phr1
uvde
B
CT value
Hours
0 8 14 22
xpc
ddb1
C
CT value
Hours
0 8 14 22
rad14
rad2
xpc
rad23
cul4
ddb1
csb
D
CT value
Hours
0 4 8 14 18 22 26
mag1
mgt1
